# Multi-Omics Analysis Provides Insights into Developmental Tepal Coloration in *Gloriosa superba* ‘Passion Flame’

**DOI:** 10.3390/plants15020235

**Published:** 2026-01-12

**Authors:** Xinyi Zhou, Kuang Sheng, Tong Wu, Liangsheng Zhang, Yuwei Liang, Xiaojun Chang

**Affiliations:** 1 Genomics and Genetic Engineering Laboratory of Ornamental Plants, College of Agriculture and Biotechnology, Zhejiang University, Hangzhou 310027, China; 2 Biotechnology and Germplasm Resource Institute, Yunnan Academy of Agricultural Sciences, Kunming 650204, China; 3 National Key Laboratory for Development and Utilization of Forest Food Resources, Zhejiang A&F University, Hangzhou 311399, China; 4 Provincial Key Laboratory for Non-Wood Forest and Quality Control and Utilization of Its Products, Zhejiang A&F University, Hangzhou 311399, China

**Keywords:** flower color, ornamental plant, anthocyanin, transcriptome, metabolome

## Abstract

*Gloriosa superba* ‘Passion Flame’ (flame lily) is a distinctive ornamental plant characterized by its striking floral structure and vivid coloration. During flower development, flame lily tepals undergo a pronounced color transition from green at the bud stage to bright red with a yellow base at maturity, providing an excellent system for studying flower pigmentation in monocots. Here, we applied a multi-omics approach to examine metabolite accumulation and gene expression dynamics across four stages of flower development. Metabolomic profiling identified 240 flavonoids and four anthocyanins, among which pelargonidin-3-O-glucoside showed the highest relative abundance among red pigmentation. Transcriptome analysis revealed that seven key anthocyanin structural genes showed strong correlations with anthocyanin accumulation. In parallel, several chlorophyll degradation genes, including *GsSGR* and *GsPPH*, were upregulated during tepal maturation, suggesting transcriptional activation of chlorophyll degradation pathways concurrent with pigment accumulation. Co-expression network analysis further identified *GsMYB75* and *GsMYB114* as temporally distinct regulators associated with anthocyanin biosynthesis, acting together with bHLH, NAC, and AP2/ERF transcription factors. This study provides new insights into the pigment regulation in *G. superba* ‘Passion Flame’ and offers candidate regulatory components for future functional studies and the improvement of ornamental traits in monocotyledonous plants.

## 1. Introduction

*Gloriosa superba* is a prized ornamental species renowned not only for its striking flame-like flowers but also as the primary natural source of colchicine, a compound widely used in traditional medicine and pharmaceuticals [1,2]. The unique floral structure of flame lily, characterized by backward-curling tepals with wavy edges, combined with vibrant colors such as bright red and yellow, makes it an exquisite ornamental plant. As an emerging monocot ornamental plant, the aesthetic features of the flame lily merit in-depth exploration.

Flower coloration is a key trait that enhances both the ornamental appeal and economic value of plants. In addition to its aesthetic importance, pigmentation plays essential roles in attracting pollinators, protecting against environmental stresses, and mediating plant–environment interactions. Over the years, extensive research has focused on the biochemical and genetic pathways underlying flower coloration in various species. The diverse colors of flowers are primarily determined by the synthesis, accumulation, and modification of pigments such as flavonoids, carotenoids, and chlorophylls [3,4,5]. In the flavonoid biosynthesis pathway, anthocyanins are responsible for the pink, red, purple, and blue colors of flowers and other plant organs. Chalcones and aurones impart deep yellow colors, while flavones and flavonols are faint yellow or almost colorless. The flavonoid synthesis pathway of in plants has been largely elucidated and represents a branch of the phenylpropane metabolic pathway. The early stage involves the conversion of phenylalanine to 4-coumaroyl-CoA through reactions catalyzed by phenylalanine ammonia-lyase (*PAL*), cinnamic acid-4-hydroxylase (*C4H*), and 4-coumaric acid coenzyme A ligase (*4CL*) [6]. In the middle stage, 4-coumaroyl-CoA is converted to three different dihydroflavonols by chalcone synthase (*CHS*), chalcone isomerase (*CHI*), flavanone 3-hydroxylase (*F3H*), flavonoid 3′-hydroxylase (*F3′H*), and flavonoid 3′,5′-hydroxylase (*F3′5′H*), flavone synthase (*FNS*) and flavonol synthase (*FLS*) [6,7]. In the late stage, three different anthocyanidins are formed by the actions of dihydroflavonol-4-reductase (*DFR*) and leucocyanidin dioxygenase/anthocyanin synthase (*ANS*), followed by the formation of stable anthocyanins through a series of glycosylation and methylation reactions [6,8]. Transcription factors involved in regulating anthocyanin biosynthesis include MYB, bHLH and WD40, which modulate anthocyanin synthesis by either activating or repressing the expression of structural genes [9]. In addition, other TFs, such as NACs, WRKY, *HY5*, and ERFs, are involved in regulating flavonoid biosynthesis [10,11]. This regulatory network involves multiple classes of transcription factors acting at different levels of the flavonoid biosynthetic pathway.

Extensive research has been conducted on anthocyanin biosynthesis in various plants. For example, Yin et al. (2021) reported that the transcription factor *LvMYB5* can activate the *ANS* gene promoter to regulate anthocyanin biosynthesis in lily (*Lilium* Spp.) flowers [12]. Similarly, in carnation (*Dianthus caryophyllus*), one *MYB* is upregulated in petal margins alongside other MYBs, bHLHs, and *WRKY44*, promoting the expression of downstream DFR and ANS and contributing to red margin formation [13]. Research on lisianthus (*Eustoma grandiflorum*) has demonstrated that *MYB32a/b*, *MYB8b*, *CHSa*, *ANSa/b* and *F3′5′Ha/b* work together to regulate blue and violet flower coloration through the modulation of anthocyanin biosynthesis [14]. However, most of these studies have focused on model plants or widely cultivated ornamental species.

In addition to pigment biosynthesis, the dynamic balance between pigment accumulation and degradation is critical for determining the final color phenotype of flowers. Many flowering plants contain chlorophyll during early developmental stages; however, as the flower matures, chlorophyll degrades, facilitating the emergence of bright and distinctive hues. The chlorophyll metabolic pathway is well characterized and involves chlorophyll biosynthesis, interconversion between chlorophyll a and b (chlorophyll cycle), and degradation [15,16]. Molecular studies on chlorophyll degradation have been conducted in species such as lily [17], carnation [18] and chrysanthemum (*Chrysanthemum morifolium*) [19], yet the regulation of chlorophyll degradation in flame lily tepals remains unclear.

With the rapid advancement of plant biology, the integration of transcriptomic and metabolomic approaches has become a powerful tool for studying horticultural plants. Previous integrative studies have begun to elucidate the molecular basis of tepal coloration in *Gloriosa*. Sun et al. (2023) investigated anthocyanin accumulation during tepal development in *G. superba* ‘Rothschildiana’ and showed that cyanidin- and pelargonidin-based anthocyanins are the major contributors to red coloration [20]. More recently, Sun et al. (2025) extended this integrative approach to multiple *Gloriosa* varieties and demonstrated that coordinated regulation of anthocyanins and carotenoids underlies diverse tepal color phenotypes [21]. However, studies addressing the molecular basis of tepal coloration in *Gloriosa superba* remain limited, and a comprehensive analysis integrating flavonoid biosynthesis with chlorophyll degradation is still needed to fully understand the metabolic interplay underlying its distinctive color pattern. The recent availability of genomic resources for *G. superba* [1] has provided a foundation for more systematic analyses of gene expression dynamics and regulatory relationships during flower development. The objective of this study was to elucidate the molecular mechanisms governing developmental tepal coloration in *Gloriosa superba* ‘Passion Flame’ by integrating metabolomic and transcriptomic analyses across four developmental stages, with emphasis on the interplay between flavonoid biosynthesis and chlorophyll degradation. By establishing an integrated regulatory framework, this study provides a comprehensive view of tepal pigmentation dynamics and offers candidate targets for future functional studies and ornamental breeding.

## 2. Results

### 2.1. Metabolomics Analysis of Gloriosa superba ‘Passion Flame’ Tepals

**Figure 1:**
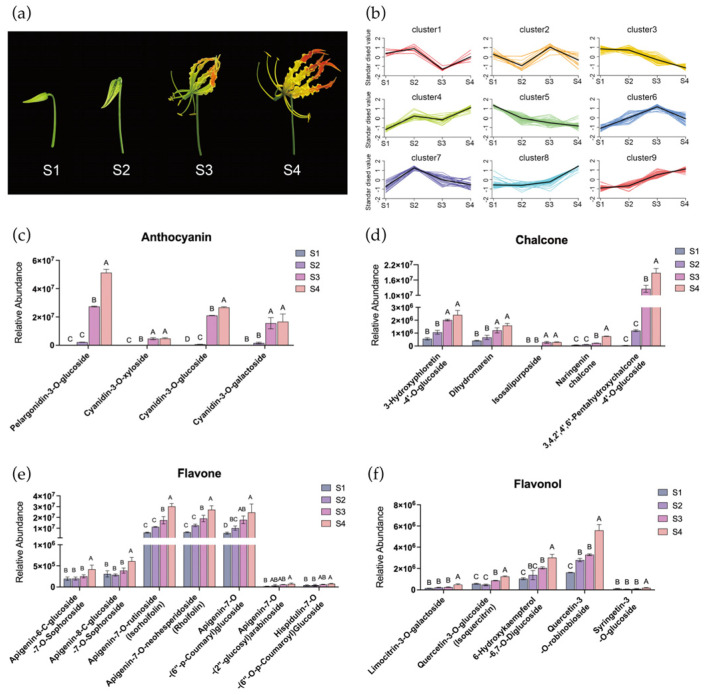
Identification and quantification of pigments in tepals of *Gloriosa superba* ‘Passion Flame’. (**a**) *Gloriosa superba* ‘Passion Flame’ flowers on the bud stage (S1), initial opening stage (S2), turning stage (S3), and mature stage (S4). (**b**) *K*-means analysis of metabolites in *G. superba* ‘Passion Flame’ tepals from S1 to S4, with flavonoids in subclass 8 and 9 were selected. The relative abundance of selected anthocyanin (**c**), chalcone (**d**), flavone (**e**) and flavonol (**f**) in flame lily tepals from S1 to S4. The data were presented as means ± SD. Different letters indicate statistically significant differences based on one-way ANOVA followed by Tukey’s multiple comparison test (*p* < 0.05).

Based on phenotypic features and color changes, we divided flower development and coloration into four distinct stages: bud stage (S1), initial opening stage (S2), turning stage (S3), and mature stage (S4) (Figure 1a). Visible tepal coloration started at the turning stage (S3) and was completed by the mature stage (S4). To investigate metabolite dynamics during flower development and coloration, we analyzed metabolomic profiles of tepals at four stages (S1–S4). In total, 769 metabolites with known structures were detected and quantified in flame lily tepals by UPLC-MS/MS technology. Principal component analysis (PCA) of the metabolic quantification from the four development stages showed that all biological replicates were grouped together, which indicates a good correlation between replicates and the high reliability of our data (Figure S1).

Flavonoids, as the predominant pigment molecules in plants, were extensively characterized in flame lily tepals. A total of 240 different flavonoid metabolites were identified and classified into eight categories, including 11 chalcones, 34 flavanones, 10 flavanonols, 83 flavones, 71 flavonols, 15 flavanols, four anthocyanidins and 12 other flavonoids (Table S1). Our data reveals that the skeletons of most flavonoids in flame lily tepals include kaempferol, quercetin, and luteolin. Moreover, the prevalent type of flavonoids in flame lily tepals consists of O-glycosides, with a limited presence of C-glycosides. It is worth noting that carotenoids were not profiled in this study, as the untargeted metabolomics approach focused primarily on flavonoid and phenylpropanoid compounds.

### 2.2. Quantitative Analysis of Anthocyanins in the Gloriosa superba ‘Passion Flame’ Tepals

To gain a deeper understanding of flavonoid metabolites contributing to flame lily tepal coloration, all the metabolites were grouped using *K*-means clustering based on their relative abundance pattern changes, resulting in nine distinct clusters (Figure 1b). Cluster 8 and 9 showed strong positive correlations with tepal pigmentation, encompassing a total of 58 flavonoids (Table S2), including four anthocyanins, five chalcones, six flavonols and seven flavones. Anthocyanin accumulation is responsible for the vivid red coloration observed in mature tepals. In this study, we detected four different anthocyanins in flame lily, including pelargonidin-3-O-glucoside (Pg3G), cyanidin-3-O-glucoside (Cy3G), cyanidin-3-O-galactoside (Cy3Gal) and cyanidin-3-O-xyloside (Cy3Xyl). As shown in Figure 1c, anthocyanin levels began to increase at S2 and showed a significant increase at S3, reaching their peak at S4. Specifically, the relative abundance of pelargonidin as the source of orange and red color was significantly higher than other three cyanidin-derivatives. This dominant presence of pelargonidin at S4 likely underlies the reddish-orange coloration characteristic of mature flame lily tepals. We identified several chalcones within the flavonoid pathway as potential contributors to the yellow pigmentation observed at the tepal base and margins, including naringenin chalcone, dihydromarein, 3-hydroxyphloretin-4′-O-glucoside, isosalipurposide, and 3,4,2′,4′,6′-pentahydroxychalcone-4′-O-glucoside. Although carotenoids were not assessed in this study, the accumulation pattern of isosalipurposide, which begins at S3 and peaks at S4 (Figure 1d), coincides with the emergence of yellow tones. This suggests a possible role for flavonoid-derived chalcones in yellow tepal coloration.

In addition, as shown in Figure 1e, the relative abundance of apigenin-7-O-rutinoside, apigenin-7-O-neohesperidoside and apigenin-7-O-(6′-p-Coumaryl) glucoside were significantly higher among the flavones and continuously increased throughout the flower development process. Among the flavonols, quercetin-3-O-robinobioside, quercetin-3-O-glucoside, limocitrin-3-O-galactoside and 6-hydroxykaempferol-6,7-O-diglucoside were significantly abundant at S4 (Figure 1f). These flavonoids may act as accessory pigments contributing to the coloration of flame lily tepals. Taken together, our results suggest that Pg3G and isosalipurposide are the primary pigments responsible for the red and yellow coloration among the flavonoids in flame lily tepals, respectively.

### 2.3. Transcriptome Analysis of the Tepals of Gloriosa superba ‘Passion Flame’

**Figure 2:**
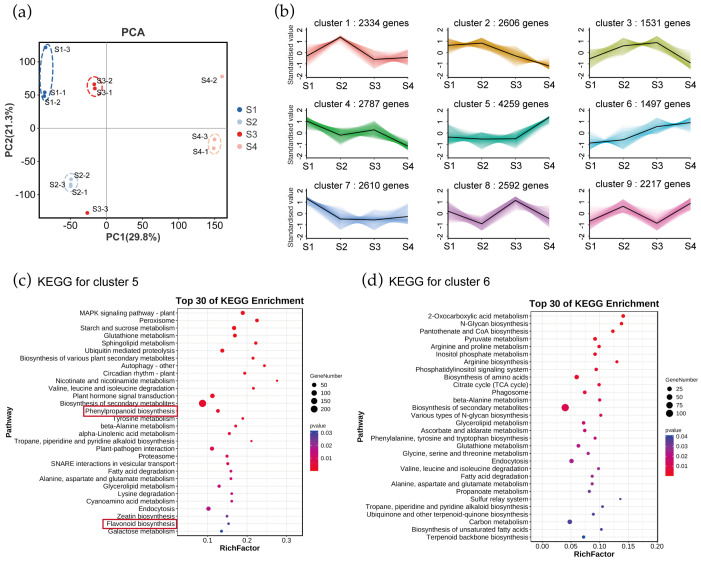
Transcriptome analysis of genes in *Gloriosa superba* ‘Passion Flame’. (**a**) PCA at four different development stages based on RNA-seq data. -1, -2, -3 refer to the three replicates. (**b**) Expressed genes were clustered into nine expression patterns through *K-means* analysis of transcriptome in *G. superba* ‘Passion Flame’ tepals from S1 to S4. KEGG pathway enrichment of genes involved in (**c**) cluster 5 and (**d**) cluster 6 showed the top 30 pathways with the most significant enrichment. Red rectangle highlights the anthocyanin-related biosynthesis pathways.

To identify key candidate genes for tepal color transitions, we analyzed RNA-seq data from flame lily tepals at four developmental stages. The *Gloriosa superba* ‘Passion Flame’ was used as the reference genome, onto which 88.83–90.48% of the clean reads were mapped. Among the mapped reads, the uniquely mapped reads accounted for 67.21–76.46%, and 13.03–23.57% were aligned to multiple loci (Table S3). These mapping statistics indicate that the RNA-seq data are of sufficient quality for further analysis. Based on PCA, samples suitable for further investigation were selected and outliers were removed. The first PC (29.8%) separated mature stage samples from other three groups of samples, demonstrating that the mature stage was the most important stage throughout the developmental periods (Figure 2a). Overall, both the metabolome and transcriptome showed significant developmental specificity during different stages of flower development in flame lily. Parallel to our analysis of metabolites, we performed *K*-means clustering analysis on the transcriptome data to identify changes in gene expression. This analysis categorized the genes into nine clusters based on their expression patterns from S1 to S4 (Figure 2b). Among them, clusters 5 and 6 showed an up-regulated trend in gene expression and a positive correlation with the coloration of the flame lily, and 4259 and 1497 genes were grouped into cluster 5 and 6, respectively (Table S4). These selected genes are potentially essential for tepal color expression in flame lilies.

To understand their biological functions and gene interactions, we annotated selected genes in cluster 5 and 6 using the KEGG database. The KEGG metabolic pathway analysis using *Q*-value < 0.05 revealed that the genes in clusters 5 and 6 were significantly enriched in various metabolic processes. For genes in cluster 5, significantly enriched pathways were MAPK signaling pathway plant, peroxisome, starch and sucrose metabolism, glutathione metabolism, sphingolipid metabolism and ubiquitin mediated proteolysis (Figure 2c). Genes in cluster 6 were enriched in pathways such as 2-Oxocarboxylic acid metabolism, N-Glycan biosynthesis, pantothenate and CoA biosynthesis, pyruvate metabolism, arginine and proline metabolism, inositol phosphate metabolism and arginine biosynthesis (Figure 2d). Notably, genes in cluster 5 were enriched in anthocyanin-related pathways including the phenylpropanoid process and flavonoid biosynthesis, whereas genes in cluster 6 were not. This indicates a closer association of genes in cluster 5 with anthocyanin synthesis and tepal coloration in flame lilies.

### 2.4. Important Pathway Genes for Anthocyanin Accumulation and Chlorophyll Degradation in Gloriosa superba ‘Passion Flame’ Tepals

**Figure 3:**
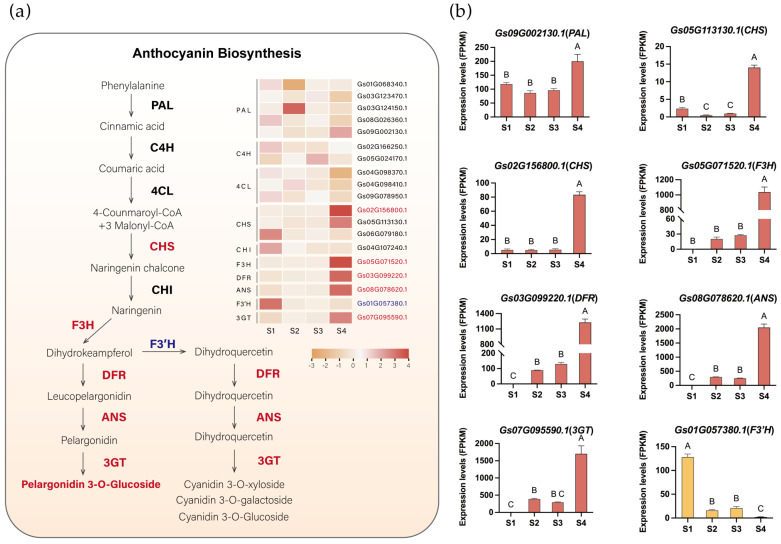
The anthocyanin biosynthesis pathway in flame lily tepals. (**a**) The proposed anthocyanin biosynthesis pathway. The expression pattern of each gene is shown in a heatmap beside. Orange indicates low expression and red indicates high expression. Potential upregulated genes are in red fonts, and downregulated genes are in blue fonts. (**b**) Histograms showing expression levels (Fragments Per Kilobase of transcript per Million mapped reads, FPKM) of important structure genes involved in anthocyanin biosynthesis. The data were presented as means ± SD. Different letters indicate statistically significant differences based on one-way ANOVA followed by Tukey’s multiple comparison test (*p* < 0.05). Note: *PAL*, Phenylalanine ammonia-lyase; *C4H*, cinnamate-4-hydroxylase; *4CL*, 4-coumarate CoA ligase 4; *CHS*, chalcone synthase; *CHI*, chalcone isomerase; *F3H*, flavanone 3-hydroxylase; *DFR*, dihydroflavonol 4-reductase; *ANS*, anthocyanidin synthase.

To identify critical genes involved in pigment metabolism in flame lily tepals, we identified anthocyanin biosynthetic pathway genes through sequence homology searches and phylogenetic analysis. A total of 21 anthocyanin-related genes were obtained, including *PAL*, *4CL*, *C4H*, *CHS*, *CHI*, *F3H*, *F3′H*, *DFR*, *ANS*, and *3GT* (Figures S2–S9). Genes with detectable expression in the transcriptome were retained for further analysis. Notably, among these retained genes, no candidate gene encoding flavonoid 3′5′-hydroxylase (*F3′5′H*) was identified. This absence is consistent with our metabolomic results, in which no delphinidin-type anthocyanins were detected, supporting the lack of lilac to blue pigmentation in flame lily. Using transcriptome data, we visualized the expression patterns of anthocyanin biosynthetic pathway structural genes in flame lily (Figure 3a, Table S5). Seven unigenes of the six enzymes were assigned to cluster 5, which showed a high correlation with the coloration of the flame lily, and their expression profiles were presented in Figure 3b. Among them, the expression levels of one *PAL* (*Gs09G002130.1*) and two *CHS* (*Gs02G156800.1*, *Gs05G113130.1*) genes increase from S1. As they are involved in the early stage of anthocyanin biosynthesis, their expression precedes the accumulation of anthocyanins. The *F3H* (*Gs05G071520.1*), along with other late-stage genes including *ANS* (*Gs08G078620.1*), *DFR* (*Gs03G099220.1*), and *UFGT* (*Gs07G095590.1*), showed a progressive increase in expression levels from S2 to S4, peaking significantly at S4 (Figure 3b), suggesting that anthocyanin accumulation started at S2. However, *F3′H* (*Gs01G057380.1*) displayed an inverse trend to the coloration changes in the tepal, peaking at S1 and down-regulated during development (Figure 3b).

**Figure 4:**
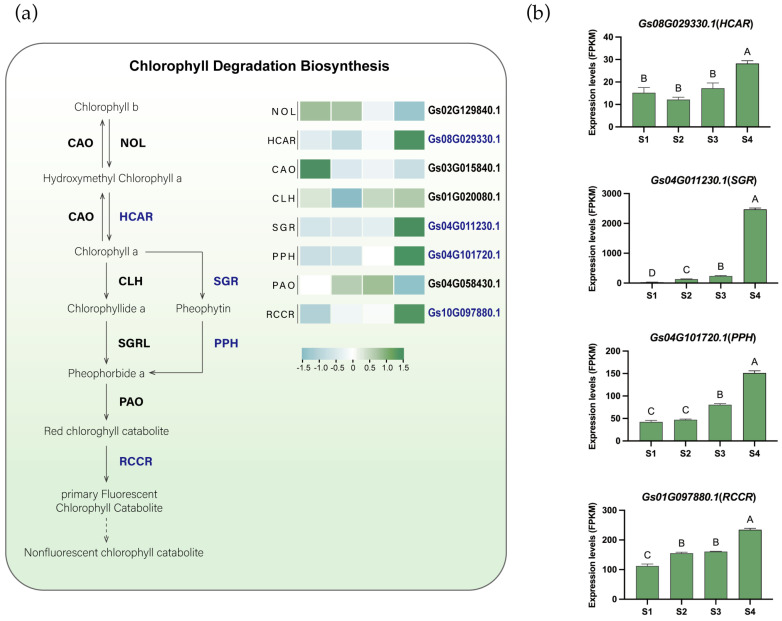
The proposed chlorophyll degradation biosynthesis pathway in flame lily tepals. (**a**) The proposed chlorophyll degradation pathway. Gene expression levels are presented in heatmaps next to the gene names. Low to high expression is indicated by a change in color from blue (low accumulation) to green (high accumulation). Potential essential genes are in blue fonts. (**b**) Histograms showing expression levels (FPKM) of potential important genes involved in chlorophyll degradation. The data were presented as means ± SD. Different letters indicate statistically significant differences based on one-way ANOVA followed by Tukey’s multiple comparison test (*p* < 0.05). Note: *NOL*, chlorophyll b reductase; *HCAR*, hydroxymethyl chlorophyll a reductase; *CAO*, chlorophyllide a oxygenase; *CLH*, chlorophyllase; *SGR*, Mg-dechelatase STAY-GREEN; *PPH*, pheophytinase; *PAO*, pheophorbide a oxygenase; *RCCR*, red chlorophyll catabolite reductase.

Another significant color change in flame lily tepals was the gradual loss of greenness. To explore whether this phenotypic change was accompanied by transcriptional changes in chlorophyll degradation metabolism, we identified and analyzed the expression patterns of key genes involved in this process, including Chlorophyllide a oxygenase (*CAO*), chlorophyll b reductase (*NOL*), chlorophyllase (*CLH*), hydroxymethyl chlorophyll a reductase (*HCAR*), STAY-GREEN (*SGR*), pheophytinase (*PPH*), pheophorbide a oxygenase (*PAO*), and Red chlorophyll catabolic reductase (*RCCR*) (Figure 4a, Figures S10–S15, Table S5). Based on *K*-means clustering analysis, genes such as *HCAR* (*Gs08G029330.1*), *SGR* (*Gs04G011230.1*), *PPH* (*Gs04G101720.1*), and *RCCR* (*Gs01G097880.1*) were grouped into Cluster 5 and showed up-regulated expression during flower development (Figure 4b), suggesting their potential involvement in chlorophyll degradation in the tepals. Chlorophyll degradation is thought to occur through two main pathways: the CLH pathway and the PPH pathway. In flame lily, the expression of *GsPPH* increased progressively during development, showing an approximately 2.8-fold increase at S4 compared with S1, whereas *GsCLH* exhibited a more moderate change (approximately 1.1-fold). In addition, the expression of *CLH* is relatively low, and *SGRL* is not expressed. These expression patterns suggest a preferential transcriptional activation of the PPH-associated chlorophyll degradation pathway during tepal development. Therefore, *GsSGR* appears to be the key rate-limiting enzyme in chlorophyll degradation and may play a crucial role in this process. The temporal upregulation of chlorophyll catabolic genes occurred concomitantly with anthocyanin accumulation, reflecting coordinated transcriptional changes in pigment-related metabolic pathways during anthesis.

### 2.5. Important Transcription Factors for Coloration of Gloriosa superba ‘Passion Flame’ Tepals

**Figure 5:**
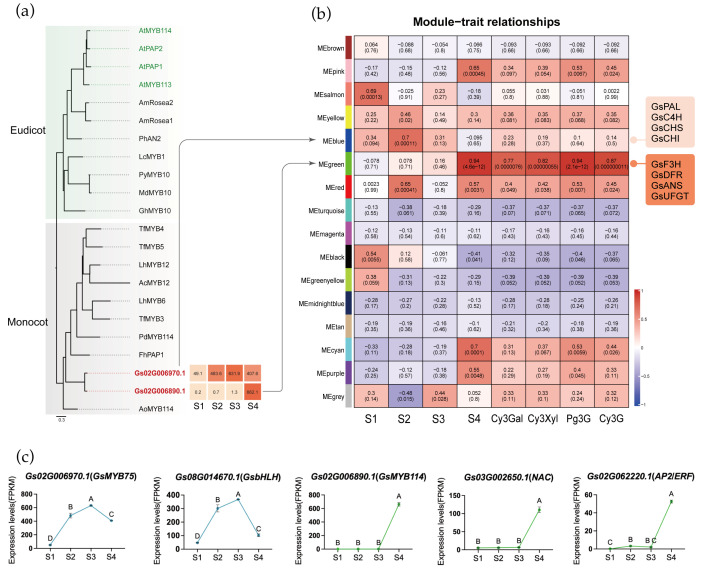
Potential transcription factors of tepal coloration biosynthesis. (**a**) Phylogenetic analysis of MYB proteins from different plants. *MYB* genes identified in flame lily were indicated by red bold fonts. The GenBank accession numbers for all reference MYB proteins are provided in Supplementary Table S6. (**b**) The relationship analysis between genes module and four development stages and four anthocyanins by WGCNA. Each row represents a module, the left four column represents four development stage, the right four column represents specific anthocyanin, respectively. The value in each cell at the row–column intersection represents the correlation coefficient between the module and the stage/anthocyanins is displayed according to the color scale on the right. The value in parentheses in each cell represents the *p*-value. (**c**) The expression profiles (FPKM) of candidate transcription factors in four stages. The data were presented as means ± SD. Different letters indicate statistically significant differences based on one-way ANOVA followed by Tukey’s multiple comparison test (*p* < 0.05).

*MYB* transcription factors play a key role in regulating structural genes involved in the anthocyanin biosynthetic pathway. To identify candidate *MYB* genes involved in anthocyanin biosynthesis in *Gloriosa superba* ‘Passion Flame’, we conducted a phylogenetic analysis using MYB transcription factors previously characterized as anthocyanin regulators in both eudicots and monocots, as well as *Arabidopsis thaliana* (Table S6). Two *MYB* genes (*Gs02G006970.1* and *Gs02G006890.1*) from flame lily clustered within the anthocyanin-related clade and exhibited distinct expression patterns during tepal development (Figure 5a). They were designated as *GsMYB75* and *GsMYB114*, respectively. *GsMYB75* exhibited consistently high expression with a peak at stage 3, while *GsMYB114* was sharply upregulated only at the mature stage (S4). These expression patterns indicated that *GsMYB75* may contribute to the early activation of anthocyanin biosynthesis, whereas *GsMYB114* is likely involved in its late stages. Promoter analysis of key structural genes revealed conserved MYB-binding motifs in the upstream regions of *GsPAL*, *GsCHS*, *GsF3H*, *GsF3′H*, *GsDFR*, *GsANS* and *Gs3GT* (Table S7), supporting a potential direct regulatory role of these MYBs in the transcriptional control of the pathway.

To further elucidate the transcriptional regulation of pigment accumulation, we conducted WGCNA, which grouped genes into 17 modules based on expression similarity (Figure 5b, Figures S16–S18). By integrating anthocyanin content as an external trait, we identified two key modules associated with candidate MYB transcription factors. *GsMYB75* was assigned to the blue module (Table S8), which also contained early-stage anthocyanin biosynthetic structural genes such as *GsPAL*, *GsC4H*, *GsCHS*, and *GsCHI*. By contrast, *GsMYB114* was located in the green module (Table S8), which showed the highest positive correlation with both anthocyanin levels and tepal maturation. The green module also included *GsF3H*, *GsDFR*, *GsANS*, *GsUFGT*, and chlorophyll degradation-related genes *GsSGR* and *GsPPH*, indicating coordinated transcriptional expression of pigment-associated pathways during tepal development. These findings suggest that the blue module is mainly involved in early pigment biosynthesis, while the green module functions during the mature stage.

**Figure 6:**
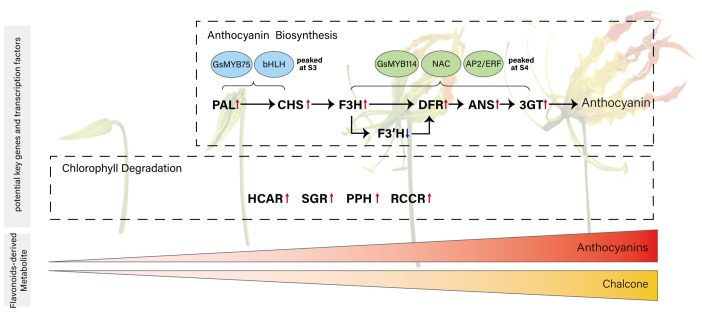
Proposed model illustrating the coordination between anthocyanin biosynthesis and transcript-level evidence of chlorophyll degradation during tepal development in *Gloriosa superba* ‘Passion Flame’. This model includes two processes: anthocyanin accumulation, which begins at the second developmental stage, and chlorophyll degradation. Genes in bold are the potential key structure genes involved in anthocyanin biosynthesis and chlorophyll degradation. Transcription factors in blue and green circles are putative regulators of early and late stages of anthocyanin biosynthesis, respectively. The color block at the bottom represents the pigment changes across developmental stages.

Among all genes in blue and green module, 182 in blue and 94 in green were annotated as transcription factors (Table S9). To further narrow down the transcription factors potentially acting with MYBs, we examined their expression patterns in a heatmap and classified them using hierarchical clustering. In both the blue and green modules, this analysis identified a single cluster whose expression pattern closely matched that of the two MYB candidates, allowing us to pinpoint a small set of transcription factors with highly similar developmental dynamics (Figure S19). After pinpointing these clusters, we further inspected the functional annotations and known gene families of the transcription factors they contained, to determine which of them might plausibly participate in pigment regulation. In the blue module, *Gs08G014670.1*, a bHLH-homolog, exhibited an expression pattern highly similar to that of *GsMYB75*, suggesting a possible MYB-bHLH partnership in activating early anthocyanin biosynthesis (Figure 5c). In the green module, *Gs03G002650.1* (*NAC*) and *Gs02G062220.1* (*AP2/ERF*) showed synchronized expression with *GsMYB114*, indicating that these factors may contribute to late-stage regulation of pigment metabolism. Although the functional roles of these TFs remain to be experimentally validated, their module assignment and co-expression with key regulators support the idea that early and late stages of color development are controlled by distinct transcription factor combinations. Building on these observations, we propose a developmental model for flame lily coloration (Figure 6) in which early-stage MYB-bHLH activity promotes anthocyanin accumulation, while late-stage *MYB*, *NAC* and *ERF* regulation reinforces pigment production and accelerates chlorophyll degradation.

The accumulation of anthocyanin initiates at S2 and reached its peak at S4, with *GsPAL*, *GsCHS*, *GsF3H*, *GsDFR*, *GsANS* and *GsUFGT* acting as central structural genes. *GsF3′H* may function as a metabolic branching point directing flux toward cyanidin production at later stages. Transcriptional regulation appears to be partitioned, with *GsMYB75* and *bHLH* driving early gene activation, and *GsMYB114* functioning alongside NAC and AP2/ERF TFs in the maturation phase. Concurrently, *GsHCAR*, *GsSGR*, *GsPPH*, and *GsRCCR* showed upregulated expression during tepal development, suggesting transcriptional activation of chlorophyll catabolic genes that may be associated with the observed loss of green pigmentation.

## 3. Discussion

### 3.1. Integrated Pigment Metabolism Underlies the Tepal Coloration in Gloriosa superba ‘Passion Flame’

The coloration of *Gloriosa superba* ‘Passion Flame’ tepals arises from the coordinated progression of anthocyanin accumulation and chlorophyll degradation during flower development, a dual process also documented in other ornamental and fruit crops such as *Paeonia suffruticosa* [22], *Litchi chinensis* [23] and *Lilium* ‘Tiny Padhye’ [17], and has been increasingly recognized as a general principle underlying plant color transitions [24]. In this study, untargeted metabolomic profiling showed that among the detected anthocyanins and flavonoid-derived yellow pigments, Pg3G and isosalipurposide exhibited the highest relative abundances, respectively. Their developmental accumulation patterns align with the red and yellow hues observed in mature tepals. Carotenoids were not detected under our current untargeted metabolomic platform, and therefore were not included in downstream pigment analyses. Consistent with this observation, isosalipurposide has also been implicated in the yellow coloration of other pale-yellow flowers such as carnations (*Dianthus caryophyllus*) [25] and tree peonies (*Paeonia suffruticosa*) [26], supporting its broader role as a contributor to yellow pigmentation in floral tissues. Most ornamentals, such as *Petunia hybrida* [27], *Rosa hybrida* [28], *Camellia japonica* [29], *Phalaenopsis*-type *Dendrobium* [30] and *Dianthus caryophyllus* [13], accumulate diverse anthocyanin profiles. Moreover, studies of other *Gloriosa* cultivars have reported the presence of approximately 60 distinct anthocyanins, indicating substantial pigment diversification across varieties [20,21]. In contrast, based on the anthocyanins detected in the present untargeted metabolomic dataset, the ‘Passion Flame’ cultivar appears to show a comparatively simpler anthocyanin profile, characterized mainly by pelargonidin- and cyanidin-derived compounds. It should be noted that untargeted metabolomics provides semi-quantitative coverage and may underestimate the full diversity of low-abundance anthocyanins. Therefore, the apparent reduction in detected anthocyanin diversity may partly reflect methodological limitations.

The absence of *F3′5′H* genes in flame lily may partially account for the lack of delphinidin-type pigments, which are commonly responsible for violet and blue colors in other flowers. Interestingly, the apparent absence of a functional F3′5′H in *Gloriosa superba* may reflect lineage-specific evolutionary specialization rather than a simple gene loss event. Previous genomic and biochemical studies have demonstrated that members of the CYP75A family in *G. superba*, including *GsCYP75A109* and *GsCYP75A110*, have been recruited into the colchicine biosynthetic pathway, where they catalyze key oxidative steps distinct from classical flavonoid hydroxylation reactions [2]. This functional divergence suggests that ancestral *CYP75A* genes may have undergone neofunctionalization, leading to a reduced or absent role in anthocyanin 3′,5′-hydroxylation. Such evolutionary trade-offs between specialized secondary metabolite pathways could constrain delphinidin biosynthesis and thereby contribute to the predominance of pelargonidin- and cyanidin-derived pigments in flame lily tepals. This hypothesis provides an evolutionary context for the observed lack of delphinidin-type anthocyanins in the ‘Passion Flame’ cultivar and warrants further comparative and functional investigation.

In parallel with pigment accumulation, a progressive loss of green coloration was observed during floral development in *Gloriosa superba* ‘Passion Flame’. To investigate whether this visual change was accompanied by transcriptional shifts in chlorophyll metabolism, we examined the expression patterns of key chlorophyll catabolic genes, including *SGR*, *PPH*, *PAO*, and *RCCR*. Several of these genes showed upregulated expression during tepal maturation, consistent with activation of the PPH-mediated chlorophyll degradation pathway at the transcript level. Among these genes, SGR is known in other species to initiate chlorophyll breakdown by destabilizing chlorophyll-protein complexes in the thylakoid membrane [31]. Functional studies in *Arabidopsis thaliana* [32], *Solanum lycopersicum* [33] and *Cucumis melo* [34] have shown that SGR promotes the early steps of chlorophyll degradation and is a major determinant of green-to-non-green color transitions. Although chlorophyll content was not directly measured in this study, the temporal expression patterns of *GsSGR* and other catabolic genes suggest that transcriptional regulation of chlorophyll breakdown pathways may be coordinated with anthocyanin biosynthesis during tepal development.

Nonetheless, due to the lack of direct pigment quantification (e.g., anthocyanin, chlorophyll, or carotenoids) our conclusions are based solely on transcriptomic and metabolomic profiles, and future targeted measurements will be required to fully resolve the contributions of different pigment pathways.

### 3.2. Developmental Regulation and Metabolic Flux Shape Pigment Composition

Anthocyanin biosynthesis in flame lily is closely linked to floral development. The major structural genes in this pathway, including *PAL*, *CHS*, *F3H*, *DFR*, *ANS* and *UFGT*, showed coordinated increases in expression during tepal maturation, consistent with the conserved anthocyanin biosynthetic cascade reported in other plant species [35,36]. The rise in *F3H* transcripts at the onset of visible coloration indicates that the entry of carbon flux into the flavonoid pathway begins early. The subsequent strong induction of *DFR*, *ANS* and *UFGT* at the mature stage matches the sharp increase in pelargonidin content.

We observed a developmental shift in the relative proportions of cyanidin- and pelargonidin-derived anthocyanins from S3 to S4 in the *Gloriosa superba* cultivar ‘Passion Flame’ (Figure 1a), indicating that anthocyanin composition changes dynamically during tepal maturation. Similar developmental fluctuations in the balance between cyanidin and pelargonidin classes have been reported in previous studies of *Gloriosa superba* ‘Rothschildiana’ [20], suggesting that such compositional shifts may be a common feature during flower development in *Gloriosa superba*. In ‘Passion Flame’, this compositional shift can be mechanistically linked to changes in F3′H expression. As F3′H converts dihydrokaempferol into dihydroquercetin, the direct precursor of cyanidin, reduced F3′H expression restricts cyanidin biosynthesis and consequently redirects metabolic flux toward pelargonidin production. Comparable relationships between F3′H activity and anthocyanin composition have been reported in other plants. In grapes (*Vitis vinifera*), reduced F3′H expression alters the ratio of cyanidin and pelargonidin derivatives during berry ripening [37], while in *Dendrobium* hybrids, weakened F3′H activity leads to enhanced pelargonidin accumulation [30]. Taken together, these results show that tepal coloration in flame lily is shaped by coordinated developmental changes in both gene expression and pathway branching. Early activation of the anthocyanin pathway establishes the initial pigment flux, while the gradual reduction in F3′H strengthens the bias toward pelargonidin production. The combination of these processes results in the dynamic color transition from pale to vivid red during tepal development.

### 3.3. Transcriptional Regulation Coordinates Pigment Biosynthesis and Chlorophyll Degradation

Transcription factors provide an additional layer of control over tepal coloration beyond the metabolic pathways themselves. Among these, two R2R3-MYB genes, *GsMYB75* and *GsMYB114*, emerged as the most likely regulators based on phylogenetic placement and co-expression patterns. Although both belong to clades known to control flavonoid biosynthesis in many species, their expression profiles indicate distinct functions during tepal maturation. *GsMYB75* accumulated mainly during the early coloring stages, when anthocyanins first begin to appear. Its strong co-expression with a bHLH gene (*Gs08G014670.1*) suggests that these two factors may form a MYB-bHLH pair similar to those known to activate early anthocyanin biosynthesis in petunia, grape, and lily. The early rise in *GsMYB75* is therefore consistent with its putative role in initiating the anthocyanin pathway.

In contrast, *GsMYB114* showed its highest expression at the mature stage and was co-expressed with NAC (*Gs03G002650.1*) and AP2/ERF (*Gs02G062220.1*) transcription factors. These gene families have been increasingly recognized as contributors to pigment regulation in various species [38]. In apple (*Malus domestica*), for instance, *MdNAC52* has been shown to activate *MdMYB9* and *MdMYB11*, promoting anthocyanin and proanthocyanidin accumulation [39]. In red-skinned pear (*Pyrus bretschneideri*), the ERF transcription factor *PyERF3* interacts with *PyMYB114* and *PybHLH3* to co-regulate anthocyanin biosynthesis [40]. Similarly, genome-wide association analysis in chrysanthemum identified NAC and AP2/ERF transcription factors as candidate regulators of anthocyanin biosynthesis and flower color variation [41]. Taken together, these findings suggest that NAC and AP2/ERF transcription factors play broader roles in pigment dynamics than previously appreciated. Late-stage co-expression patterns in flame lily suggest a similar form of regulatory integration. Notably, *GsMYB114* and the NAC gene (*Gs03G002650.1*) were co-expressed with *GsSGR*, a gene associated with chlorophyll catabolism in other species. A comparable relationship has been demonstrated in peach (*Prunus persica*), where the NAC factor *PpNAP4* activates both chlorophyll degradation genes (especially *PpSGR*) and anthocyanin biosynthetic genes (*PpANS* and *PpMYB10.1*), driving fruit coloration during maturation [42]. This raises the possibility that NAC transcription factors in flame lily may participate in coordinating anthocyanin accumulation with changes in chlorophyll-related gene expression, either independently or through interactions with MYB regulators. However, since chlorophyll content was not directly measured, this hypothesis remains tentative and warrants future physiological validation.

In addition to intrinsic developmental regulation, environmental factors, particularly light and temperature, are well-established modulators of anthocyanin biosynthesis in many plant species. Light signaling has been shown to promote anthocyanin accumulation through the action of HY5, a central regulator downstream of photoreceptors, which directly or indirectly activates R2R3-MYB transcription factors involved in flavonoid biosynthesis [43]. In several ornamental and crop plants, HY5-mediated light responses enhance the expression of MYB regulators analogous to *GsMYB75* and *GsMYB114*, thereby stimulating anthocyanin production in petals and fruits [44,45,46]. Although environmental parameters were not experimentally manipulated in this study, flame lily plants were grown under natural field conditions, where light intensity and temperature vary across developmental stages. The stage-specific activation of *GsMYB75* at early coloration stages and *GsMYB114* at later stages may therefore reflect the integration of developmental programs with ambient environmental cues. Temperature has also been reported to influence anthocyanin stability and biosynthetic gene expression, potentially contributing to the timing and intensity of tepal coloration [47,48]. While the present study focuses on transcriptional and metabolic dynamics during tepal development, future experiments incorporating controlled light and temperature treatments will be essential to disentangle environmental regulation from intrinsic developmental control and to determine whether *GsMYB75* and *GsMYB114* act as downstream nodes of light- and temperature-responsive signaling pathways in *Gloriosa superba*.

Taken together, these findings suggested a temporal division of labor among transcription factors during tepal development. An early MYB-bHLH module likely initiates anthocyanin synthesis, while a later regulatory phase involving MYB, NAC, and AP2/ERF factors strengthens pigment accumulation and may accelerates chlorophyll breakdown. A similar temporal diversification of MYB functions has been described in other monocots. In *Lilium*, for example, *LhMYB6* and *LhMYB12* regulate anthocyanin biosynthesis at different developmental stages of the petal [49]. Such a shift in regulatory control provides a plausible framework for the sequential appearance of red pigmentation and the fading of green color in flame lily, and underscores the importance of transcriptional timing in determining floral color transitions.

## 4. Materials and Methods

### 4.1. Plant Materials and Data Availability

The RNA-seq and metabolite datasets analyzed in this study were originally generated and publicly released by Liang et al. (2025) [1], and were reanalyzed in this study to investigate the regulatory mechanisms underlying tepal coloration. The *Gloriosa superba* plants of the cultivar ‘Passion Flame’ used for RNA-seq and metabolomic profiling were grown under natural field conditions in Kunming, Yunnan Province, China (25.04° N, 102.72° E). Flower samples were collected at four developmental stages defined by tepal morphology and coloration: bud stage (S1), initial opening stage (S2), turning stage (S3), and mature stage (S4) (Figure 1a).

All data used in this study were downloaded from Figshare repository (https://figshare.com/articles/dataset/_b_The_giant_genome_of_lily_provides_insights_into_the_hybridization_of_cultivated_lilies_b_/27933375) (accessed on 6 January 2026), including genome, metabolomic datasets, and twelve RNA-seq libraries (four developmental stages × three biological replicates). All datasets were reanalyzed in the present study to characterize pigment accumulation and gene expression dynamics across developmental stages. The processed data and analysis outputs generated in this study are provided in the Supplementary Materials.

### 4.2. Metabolomic Analysis

Untargeted metabolomic profiling was performed using entire tepal tissues collected at each developmental stage (S1–S4), with three biological replicates per stage. Metabolite abundance data were unit-variance scaled prior to analysis. Metabolite abundance data were unit-variance scaled prior to analysis. Unsupervised PCA and K-means clustering were performed in R (version 4.2.2; www.r-project.org) using the base functions prcomp and kmeans.

### 4.3. RNA Sequencing and Gene Expression Analysis

For transcriptomic analysis, total RNA was extracted from the entire tepal tissue at each developmental stage (S1–S4). Raw sequencing reads were quality filtered using fastp v0.23.4 [50] and aligned to chromosome-scale reference genome using HISAT2 v2.2.0 with the default parameters [51]. Cufflinks (v2.1.1) was used to calculate gene expression levels as FPKM based on the aligned RNA-seq data [52]. The resulting expression matrix was used for clustering and network analyses. K-means clustering [53] was applied to cluster genes with similar developmental expression trajectories across the four tepal stages. KEGG pathway enrichment analysis was then performed for selected gene clusters (clusters 5 and 6) using KOBAS [54] based on annotations from the Kyoto Encyclopedia of Genes and Genomes (http://www.genome.jp/kegg/) (accessed on 6 January 2026) [55]. FPKM values were used for visualization of gene expression patterns across developmental stages.

### 4.4. Identification of Tepal Color Related Genes and Transcription Factors

To confirm gene identity and distinguish closely related members within pigment biosynthesis gene families, a combined HMM-based annotation and phylogenetic analysis was performed. Hidden Markov model (HMM) profiles of pigment biosynthesis related domains were downloaded from Pfam database. Candidate family members were identified by hmmsearch in HMMER v3.4 (E-value ≤ 1 × 10^−5^) [56], using a query/reference set that included proteins from representative plant species (*Oryza sativa*, *Arabidopsis thaliana*, *Acorus tatarinowii*, *Dioscorea alata*, *Nymphaea colorata*, *Allium sativum*, *Spirodela polyrrhiza*, *Acanthochlamys bracteate*, *Zostera marina*, *Phalaenopsis equestris*) to improve the robustness of family-level identification. To further validate gene identity and resolve paralogous relationships, the identified candidate sequences were subjected to phylogenetic analysis together with functionally characterized *Arabidopsis thaliana* proteins corresponding to each pigment biosynthesis gene family. All sequences were aligned using Mafft v7.526 (default parameters) [57], and phylogenetic trees were constructed with FastTree v2.1.11 [58]. Tree visualization was performed by Chiplot (https://www.chiplot.online/) (accessed on 6 January 2026). Final gene assignments were determined based on both conserved domain architecture and phylogenetic placement relative to annotated reference genes. Transcription factors were predicted using plantTFDB v4.0 [59].

To clarify the evolutionary relationships and functional classification of MYB transcription factors involved in anthocyanin biosynthesis, a dedicated phylogenetic analysis was conducted for the MYB family. Functionally validated anthocyanin-related MYB protein sequences were retrieved from the KEGG database (https://www.genome.jp/kegg/) (accessed on 6 January 2026) [55] and used as reference sequences. The reference *MYB* genes included in the phylogenetic analysis are listed in the Supplementary Table S6. Phylogenetic reconstruction was performed using the same procedures described above.

### 4.5. Weighted Gene Co-Expression Network Analysis (WGCNA)

Gene co-expression networks were constructed using the WGCNA package in R [60], in which pairwise gene expression correlations were used to define weighted network connections. Modules were created using the default settings, with the exceptions of minModuleSize, power, and mergeCutHeight being, respectively, set to 30, 14 and 0.25. The initial clusters were merged onto eigengenes. Module eigengenes, representing the dominant expression pattern of each co-expression module, were calculated and correlated (Pearson correlation) with metabolite abundance data, including key anthocyanins quantified from metabolomic profiling, to identify modules associated with pigment accumulation during flower development. Heatmap for transcription factors within selected module was constructed based on transcriptome data using Chiplot (https://www.chiplot.online/) (accessed on 6 January 2026). To further explore regulatory mechanisms, promoter sequences (1000 bp upstream to 200 bp downstream of the transcription start site) of structural genes were scanned for MYB-binding motifs using FIMO with a *p*-value cutoff of ≤1 × 10^−4^ [61].

## 5. Conclusions

In this study, we integrated metabolomic and transcriptomic analyses to uncover the molecular basis of tepal color formation in *Gloriosa superba* ‘Passion Flame’. We demonstrated that tepal coloration results from the combined effects of anthocyanin accumulation and transcript-level evidence of chlorophyll degradation. Pg3G and isosalipurposide were the most abundant red- and yellow-associated flavonoid compounds detected in this study, respectively. Transcriptome analysis revealed that key biosynthetic genes and chlorophyll catabolic genes are differentially expressed across developmental stages. Two MYB transcription factors, *GsMYB75* and *GsMYB114*, were identified as potential regulators acting at early and late stages of anthocyanin biosynthesis, respectively, alongside co-expressed bHLH, NAC, and AP2/ERF transcription factors. These findings provide new insights into the transcriptional control of pigment dynamics in monocot flowers and offer a molecular foundation for improving ornamental traits through targeted breeding strategies. As our analyses are based on previously generated datasets, future studies incorporating targeted biochemical assays and functional validation experiments will be essential to confirm the roles of the candidate genes and refine our understanding of the molecular mechanisms underlying floral pigmentation in *Gloriosa* and related ornamental species.

## Data Availability

All raw sequencing data analyzed in this study were previously generated and deposited under the project of Liang et al. (2025) [1] (Nature Communications, DOI: 10.1038/s41467-024-55545-8). The data are publicly available from the NCBI BioProject database (accession number: PRJNA1037021). All re-analyses performed here used these public datasets. Additional processed data supporting the findings of this study are available from the corresponding author upon request.

## Supplementary Materials

The following supporting information can be downloaded at: https://www.mdpi.com/article/10.3390/plants15020235/s1, Table S1. Summary of Flavonoids contents in Flame Lily Flower; Table S2. K-means Clustering Summary for Metabolites; Table S3. RNA-seq sequencing and quality statistics of flame lily flowers; Table S4. Genes in Clusters 5 & 6 from K-means Analysis; Table S5. Genes and Expression Levels in Anthocyanin Biosynthesis and Chlorophyll Degradation; Table S6. The GenBank Accession Numbers for All Reference MYB Proteins in Figure 5; Table S7. Prediction of Cis-acting Regulatory Elements for Key Anthocyanin-related Structural Genes; Table S8. List of Genes in Green and Blue Modules; Table S9.1. Transcription Factors List in Green Module; Table S9.2. Transcription Factors List in Blue Module; Figure S1. PCA analysis of metabolic; Figure S2. Phylogenetic tree of PAL genes. Genes in *Gloriosa superba* and *Arabidopsis* are in red and green fonts; Figure S3. Phylogenetic tree of C4H genes. Genes in *Gloriosa superba* and *Arabidopsis* are in red and green fonts; Figure S4. Phylogenetic tree of 4CL genes. Genes in *Gloriosa superba* and *Arabidopsis* are in red and green fonts; Figure S5. Phylogenetic tree of CHS genes. Genes in *Gloriosa superba* and *Arabidopsis* are in red and green fonts; Figure S6. Phylogenetic tree of CHI genes. Genes in *Gloriosa superba* and *Arabidopsis* are in red and green fonts; Figure S7. Phylogenetic tree of (A) F3H genes and (B) F3’H genes. Genes in *Gloriosa superba* and *Arabidopsis* are in red and green fonts; Figure S8. Phylogenetic tree of DFR genes. Genes in *Gloriosa superba* and *Arabidopsis* are in red and green fonts; Figure S9. Phylogenetic tree of (A) ANS genes and (B) UGT78 genes. Genes in *Gloriosa superba* and *Arabidopsis* are in red and green fonts; Figure S10. Phylogenetic tree of NOL genes. Genes in *Gloriosa superba* and *Arabidopsis* are in red and green fonts; Figure S11. Phylogenetic tree of (A) HCAR genes and (B) CAO genes. Genes in *Gloriosa superba* and *Arabidopsis* are in red and green fonts; Figure S12. Phylogenetic tree of CLH genes. Genes in *Gloriosa superba* and *Arabidopsis* are in red and green fonts; Figure S13. Phylogenetic tree of SGR genes. Genes in *Gloriosa superba* and *Arabidopsis* are in red and green fonts; Figure S14. Phylogenetic tree of *PPHs* and *PAOs*. Genes in Gloriosa superba and Arabidopsis are in red and green fonts; Figure S15. Phylogenetic tree of *RCCRs*. Genes in Gloriosa superba and Arabidopsis are in red and green fonts; Figure S16. (A) Soft threshold for WGCNA. (B) Eigengene adjacency heatmap; Figure S17. (A) Sample clustering diagram. (B) Gene dendrogram obtained by hierarchical clustering with the module color indicated by the color of the row underneath. A total of 16 distinct modules were identified; Figure S18. Module membership vs. gene significance in each module from WGCNA; Figure S19. (A) Heatmap showing the expression patterns of transcription factors (TFs) within the green module identified by WGCNA. (B) Heatmap of TFs in the blue module. Red-labeled gene names indicate putative MYB transcription factors potentially involved in the regulation of anthocyanin biosynthesis in *Gloriosa superba* tepals.
