# Supplementary material for: Multi-Omics Analysis Provides Insights into Developmental Tepal Coloration in Gloriosa superba ‘Passion Flame’
Source: Plants (Basel). 2026 Jan 12;15(2):235. doi: 10.3390/plants15020235 (PMC12844690; doi:10.3390/plants15020235)
Supplement: Supplementary file 1 [file plants-15-00235-s001.zip › Supplementary_Figure.pdf]

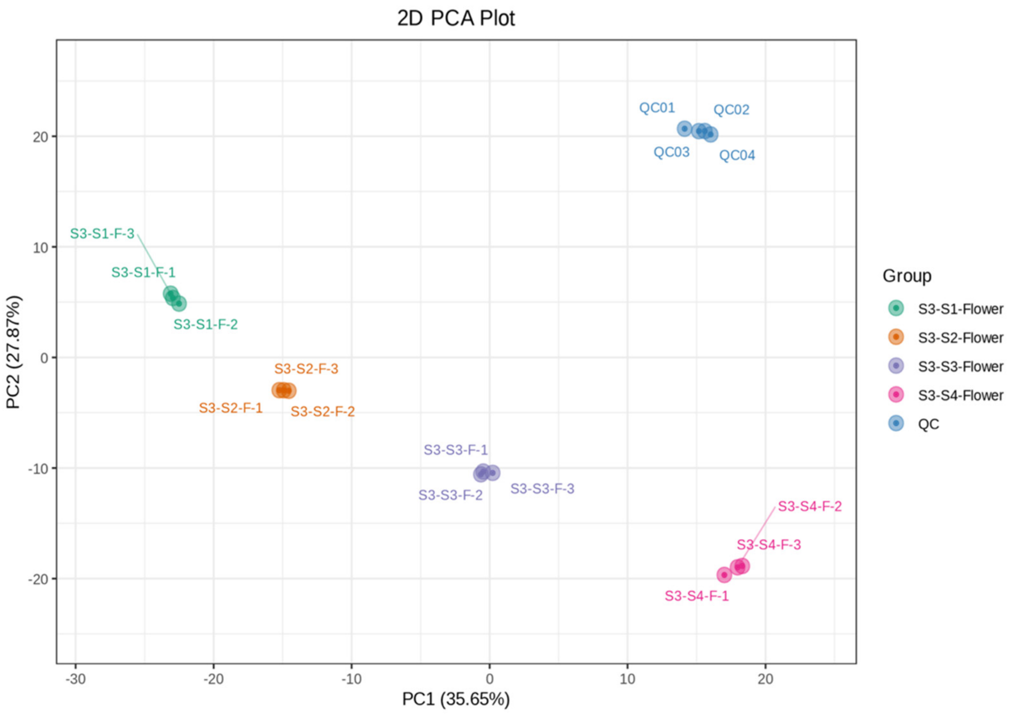

Supplementary Figure S1. PCA analysis of metabolic.

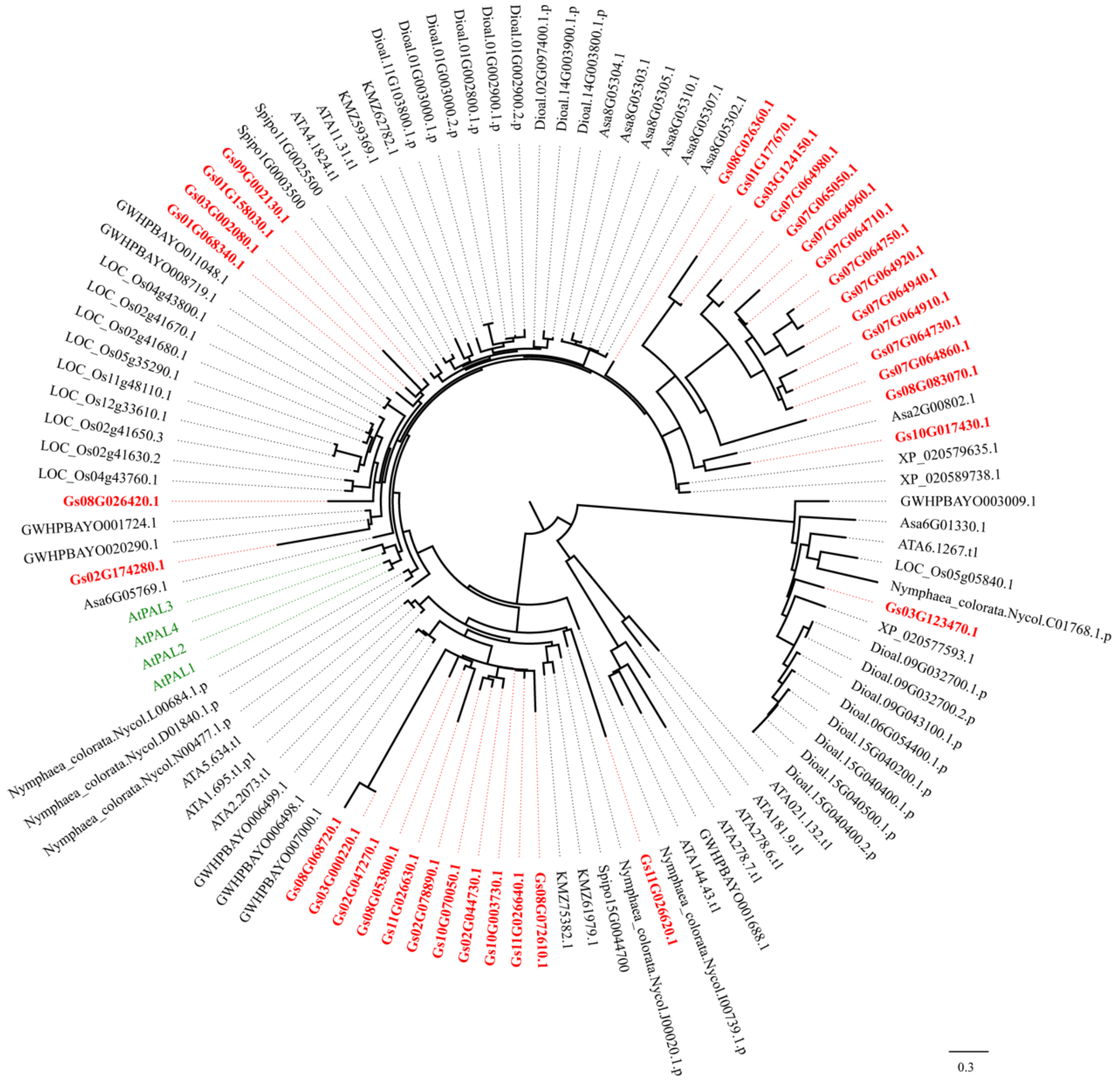

Supplementary Figure S2. Phylogenetic tree of PAL genes. Genes in *Gloriosa superba* and *Arabidopsis* are in red and green fonts.

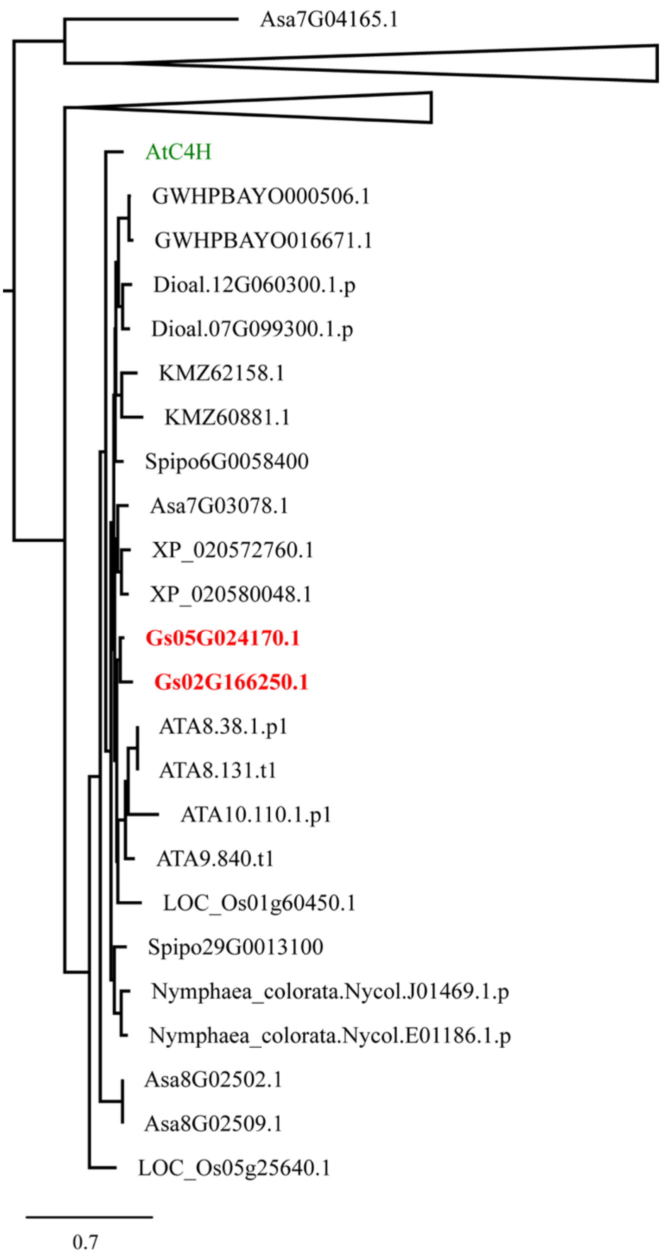

Supplementary Figure S3. Phylogenetic tree of C4H genes. Genes in *Gloriosa superba* and *Arabidopsis* are in red and green fonts.

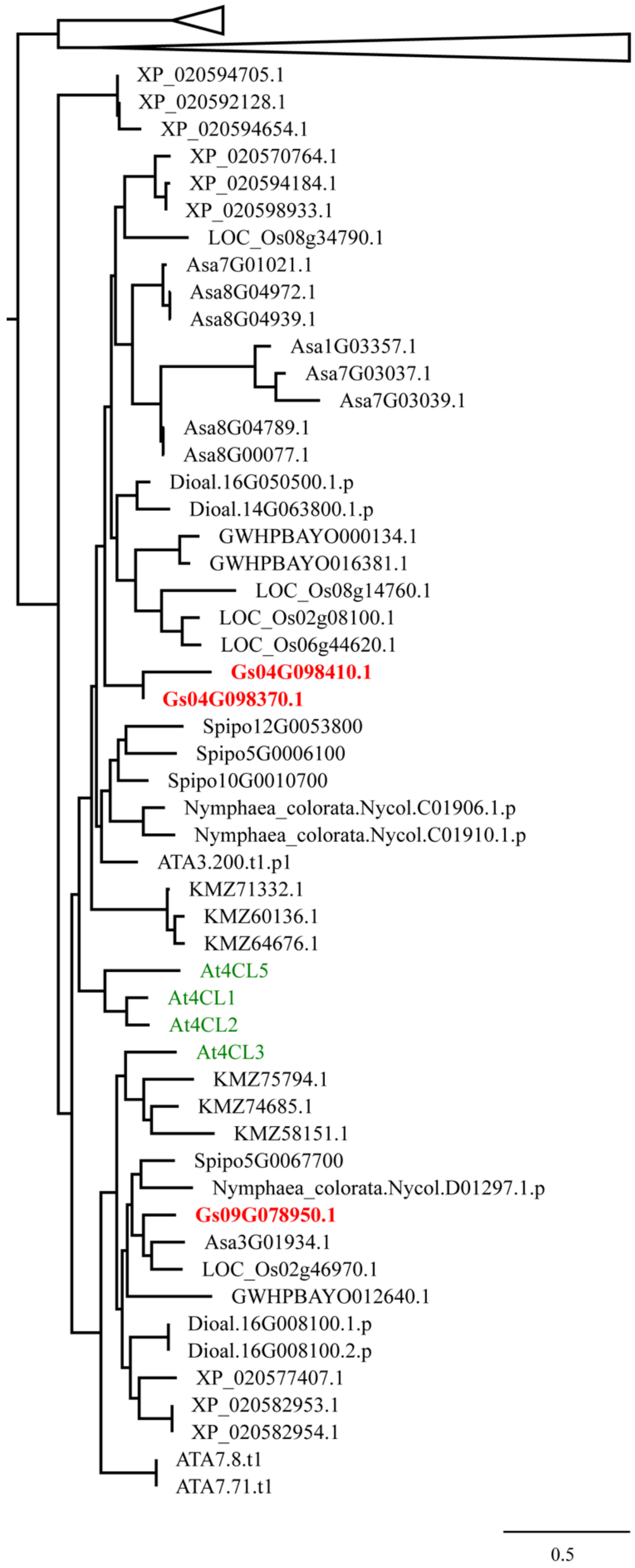

Supplementary Figure S4. Phylogenetic tree of 4CL genes. Genes in *Gloriosa superba* and *Arabidopsis* are in red and green fonts.

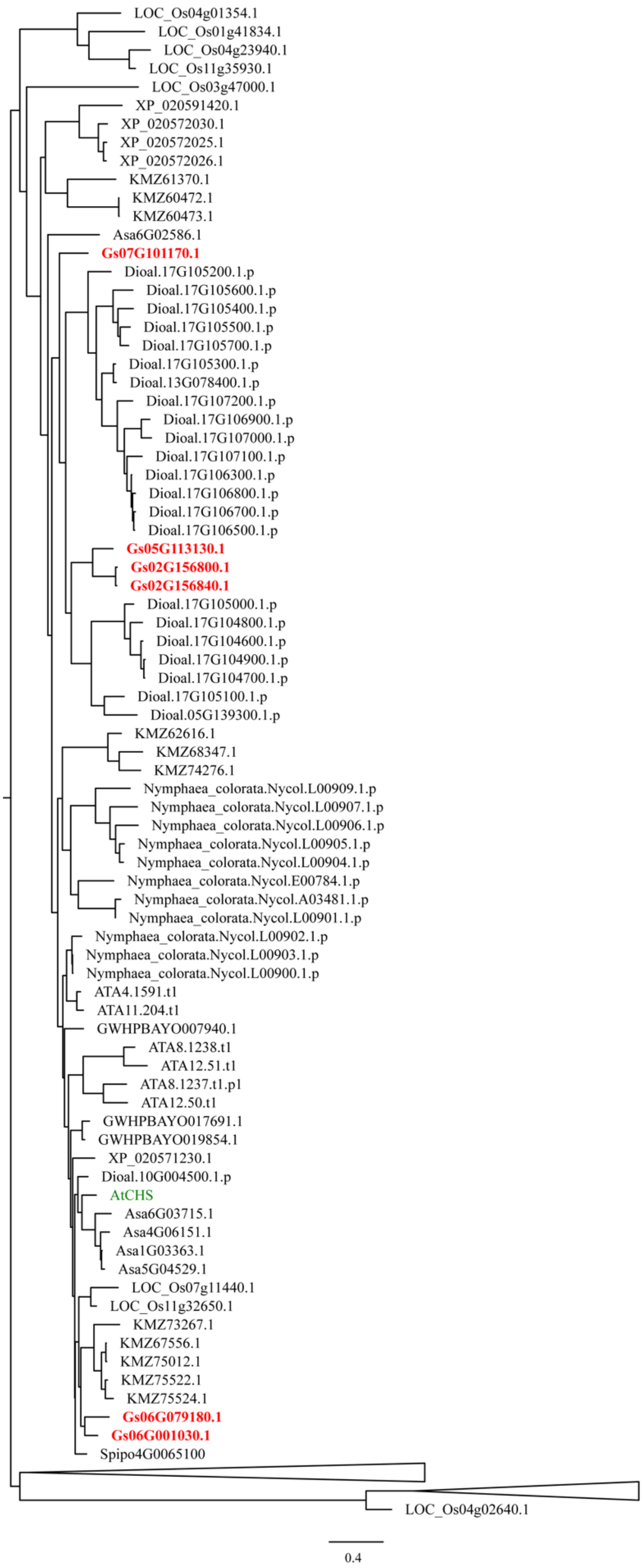

Supplementary Figure S5. Phylogenetic tree of CHS genes. Genes in *Gloriosa superba* and *Arabidopsis* are in red and green fonts.

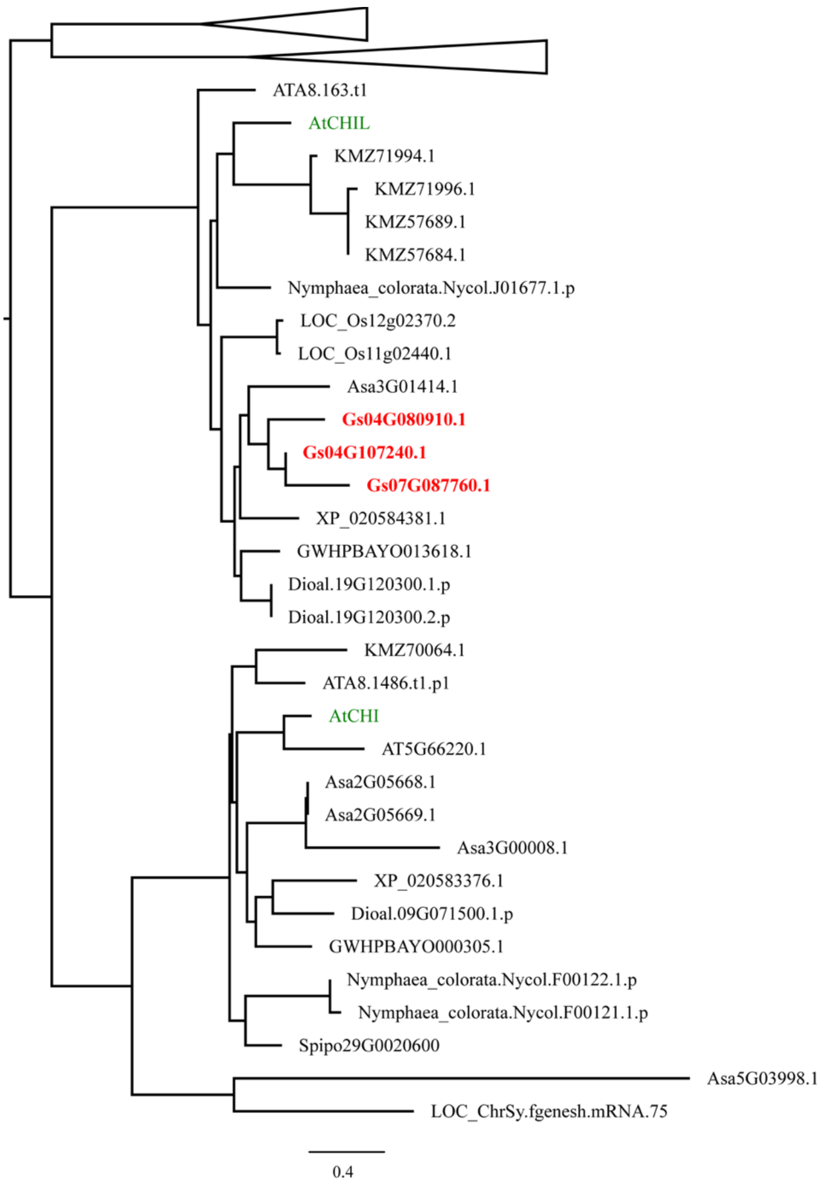

Supplementary Figure S6. Phylogenetic tree of CHI genes. Genes in *Gloriosa superba* and *Arabidopsis* are in red and green fonts.

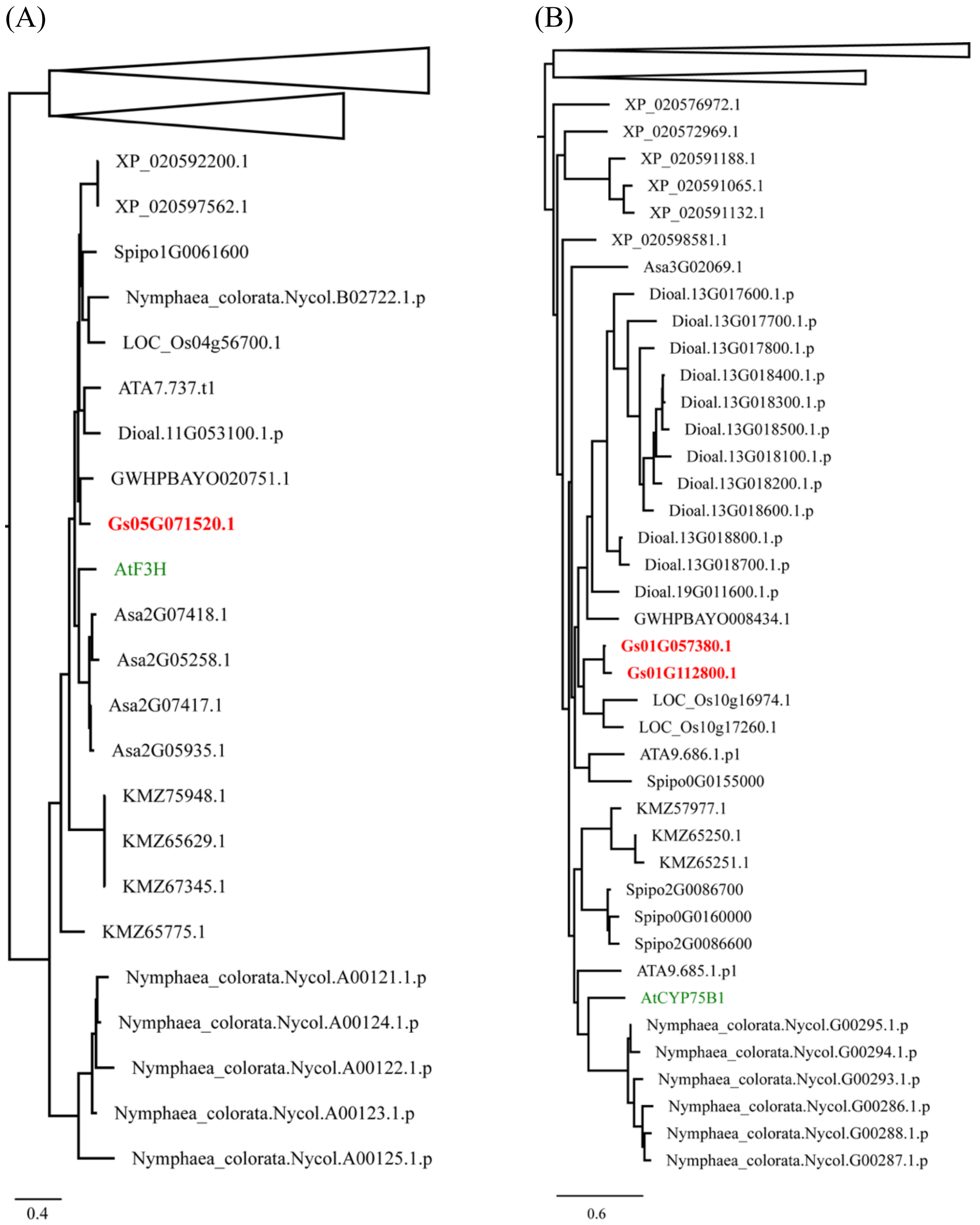

Supplementary Figure S7. Phylogenetic tree of **(A)**F3H genes and **(B)**F3’H genes. Genes in *Gloriosa superba* and *Arabidopsis* are in red and green fonts.

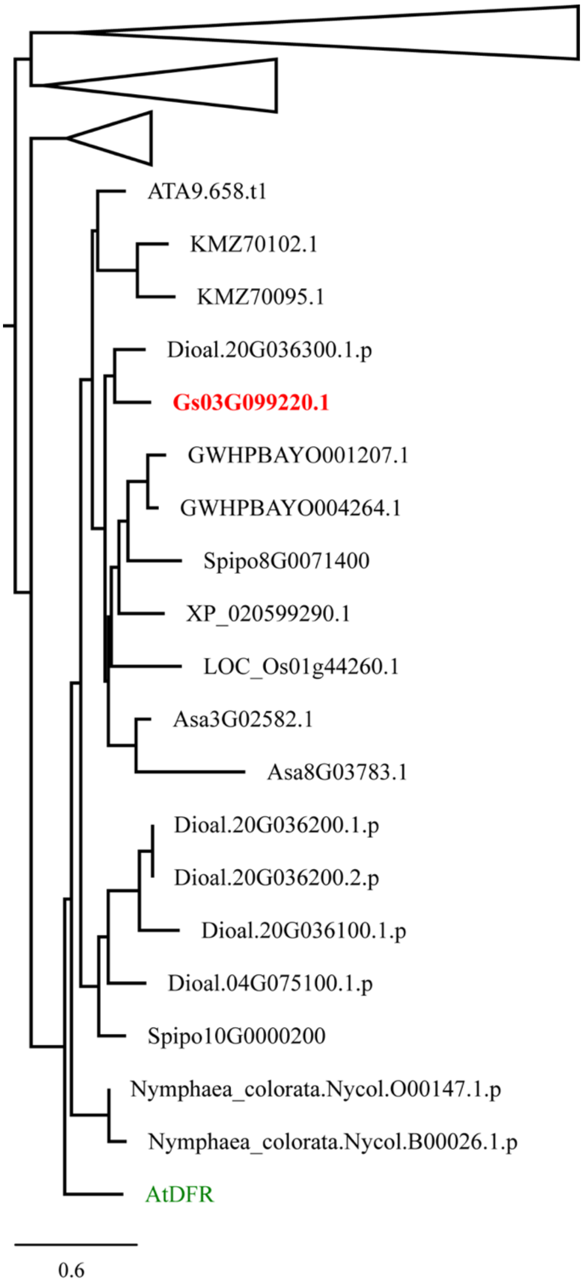

Supplementary Figure S8. Phylogenetic tree of DFR genes. Genes in *Gloriosa superba* and *Arabidopsis* are in red and green fonts.

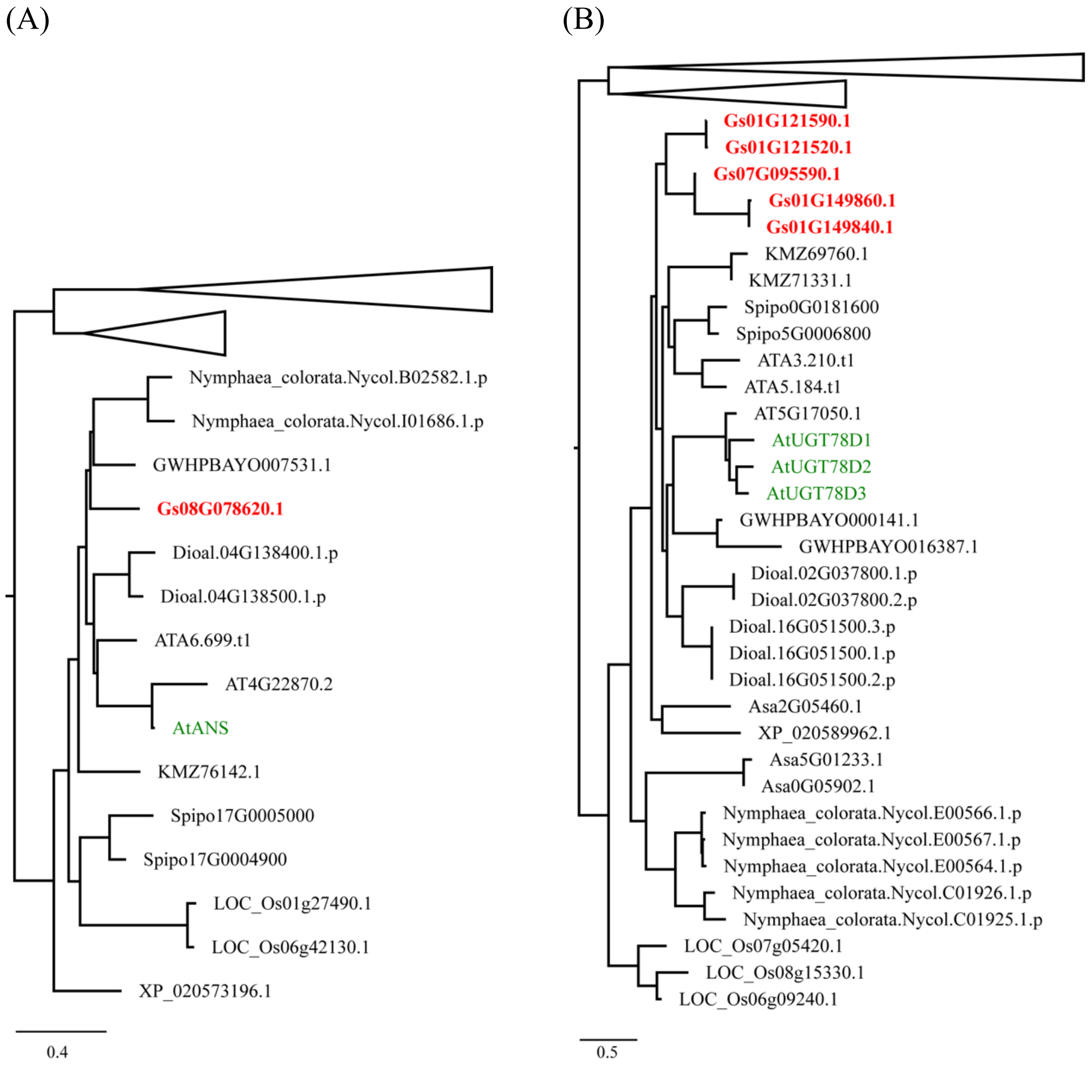

Supplementary Figure S9. Phylogenetic tree of **(A)** ANS genes and **(B)** UGT78 genes. Genes in *Gloriosa superba* and *Arabidopsis* are in red and green fonts.

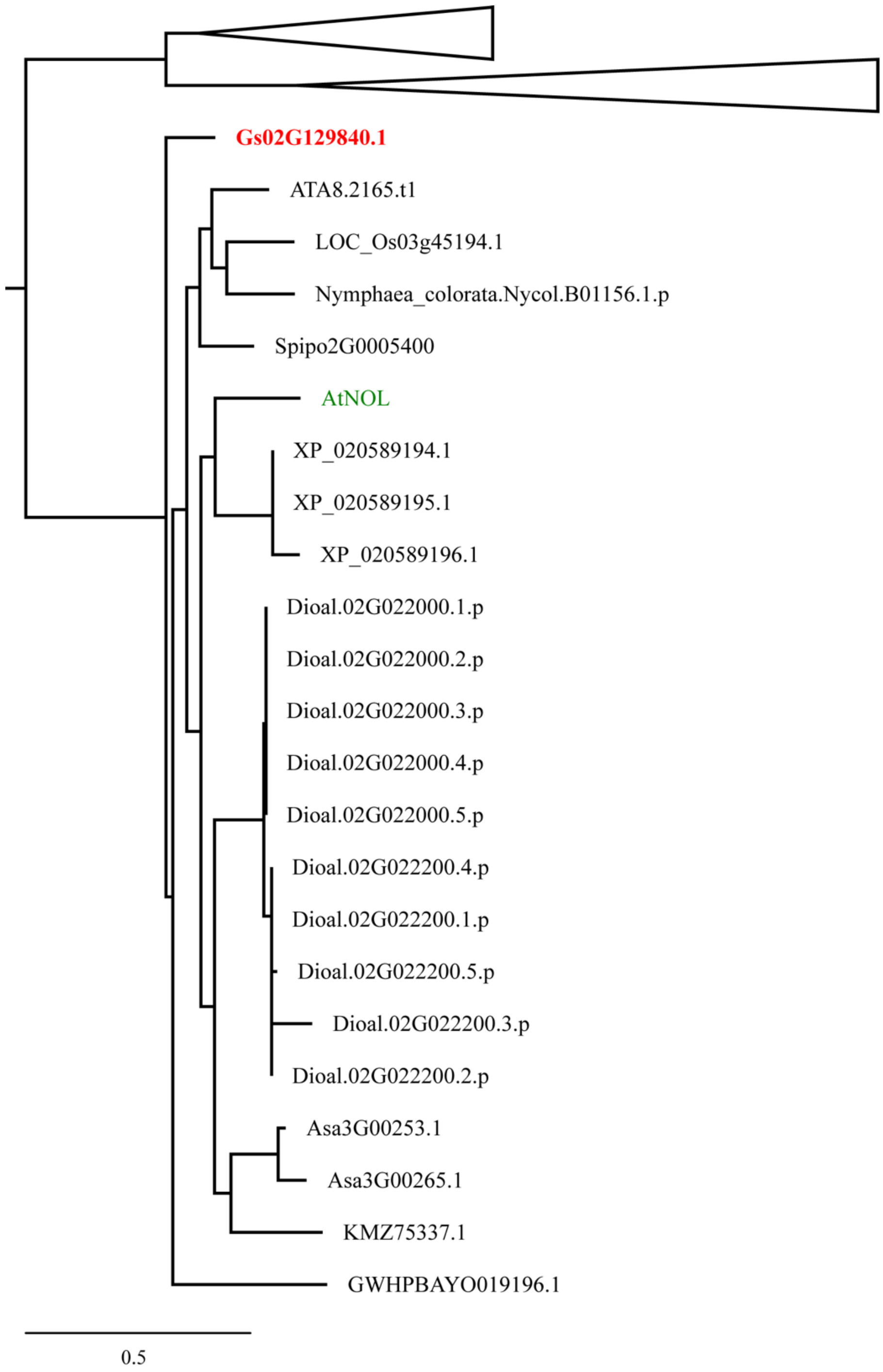

Supplementary Figure S10. Phylogenetic tree of NOL genes. Genes in *Gloriosa superba* and *Arabidopsis* are in red and green fonts.

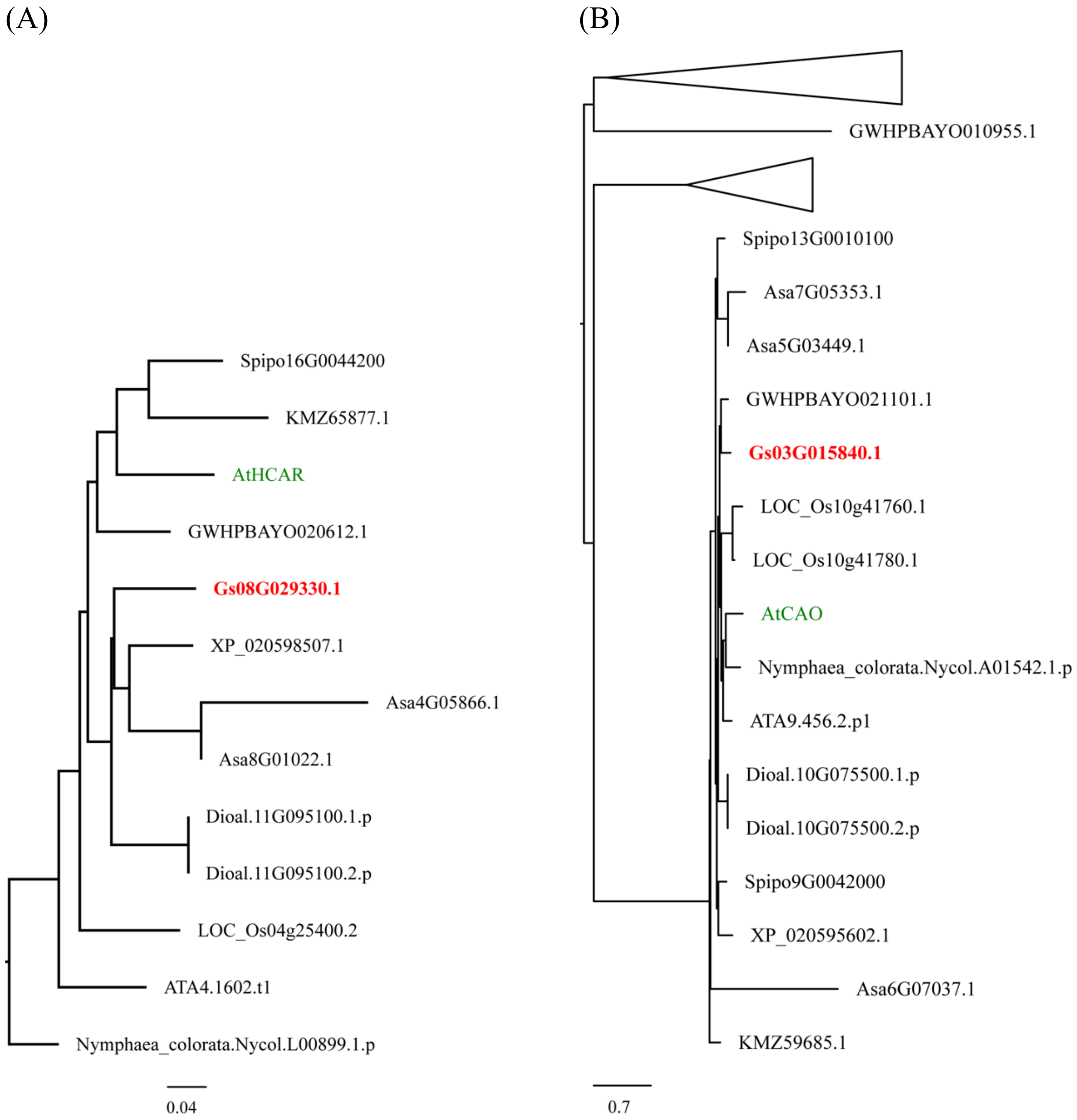

Supplementary Figure S11. Phylogenetic tree of **(A)**HCAR genes and **(B)**CAO genes. Genes in *Gloriosa superba* and *Arabidopsis* are in red and green fonts.

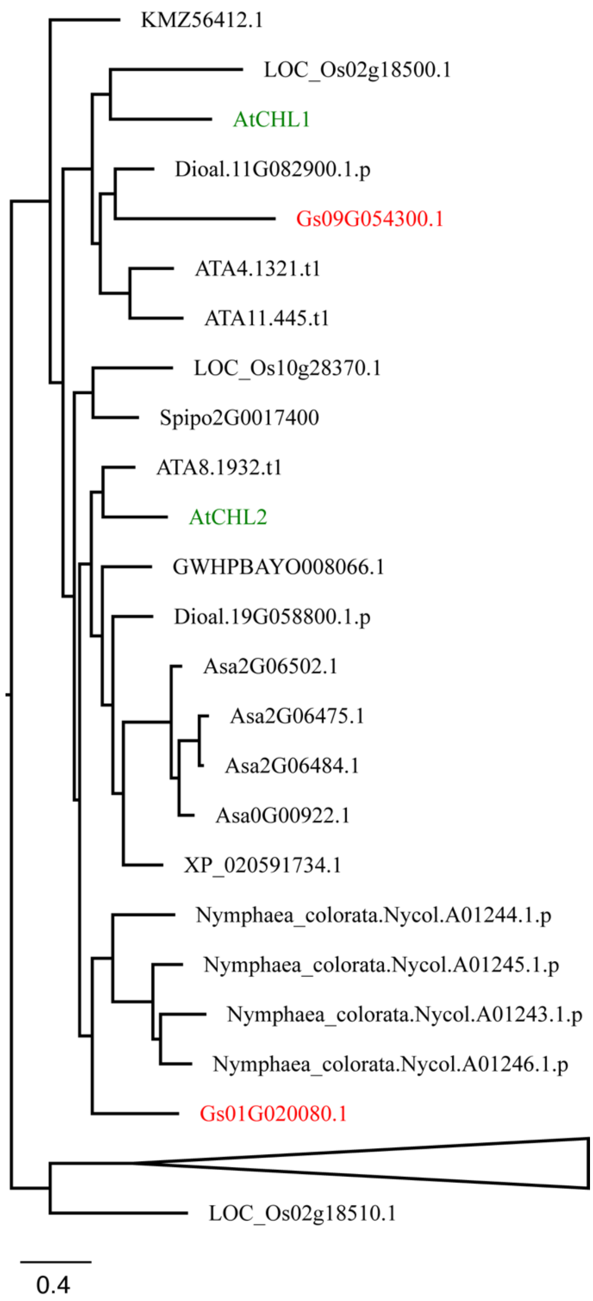

Supplementary Figure S12. Phylogenetic tree of CLH genes. Genes in *Gloriosa superba* and *Arabidopsis* are in red and green fonts.

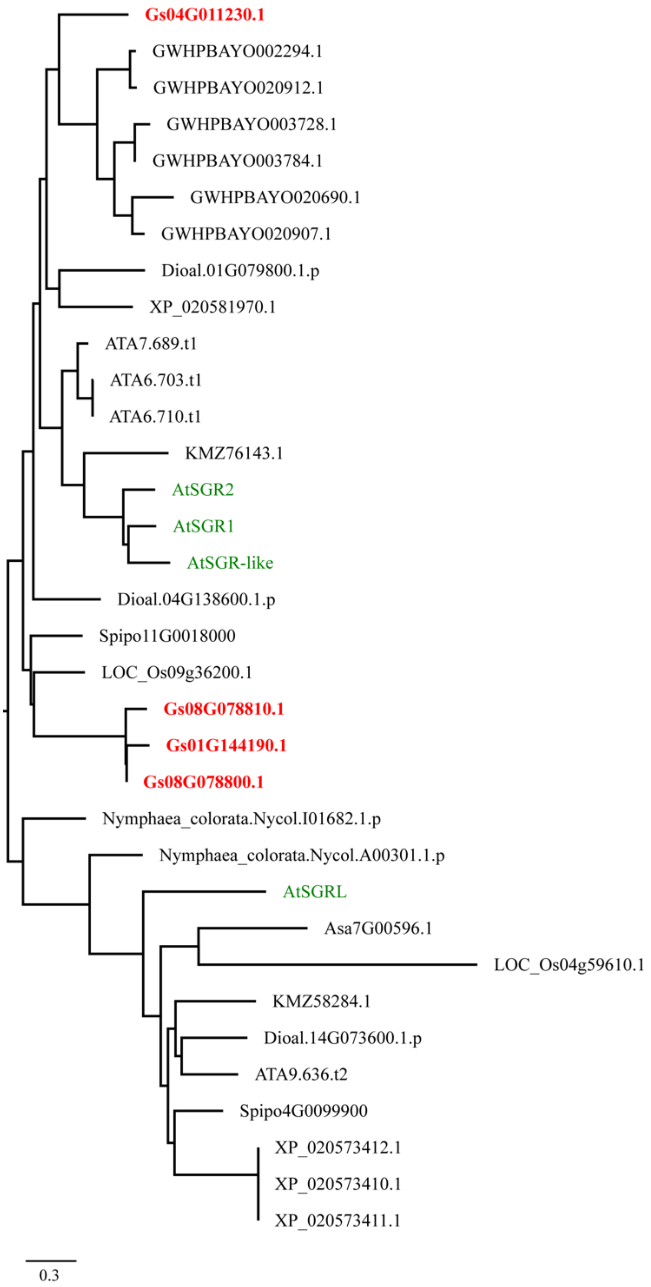

Supplementary Figure S13. Phylogenetic tree of SGR genes. Genes in *Gloriosa superba* and *Arabidopsis* are in red and green fonts.

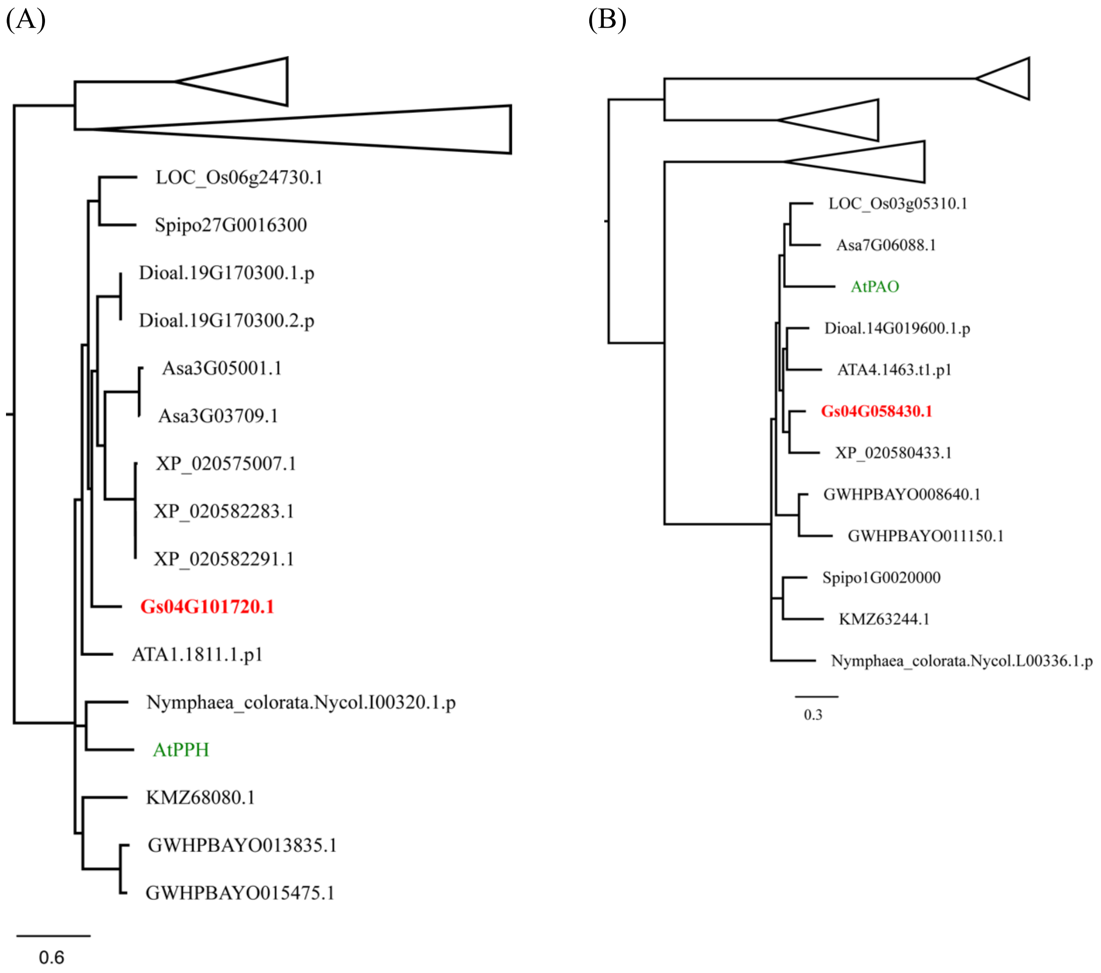

Supplementary Figure S14. Phylogenetic tree of *PPH*s and *PAO*s. Genes in Gloriosa superba and Arabidopsis are in red and green fonts.

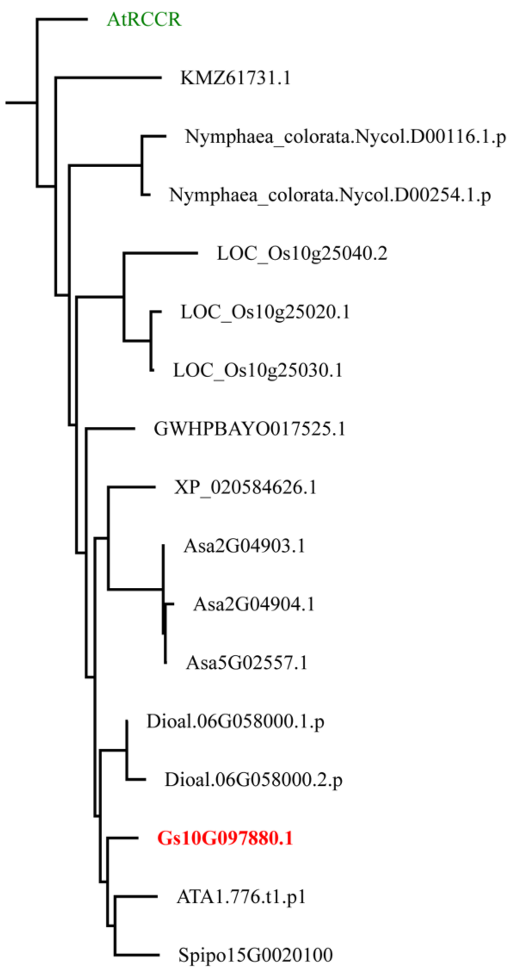

Supplementary Figure S15. Phylogenetic tree of *RCCR*s. Genes in Gloriosa superba and Arabidopsis are in red and green fonts.

(A)

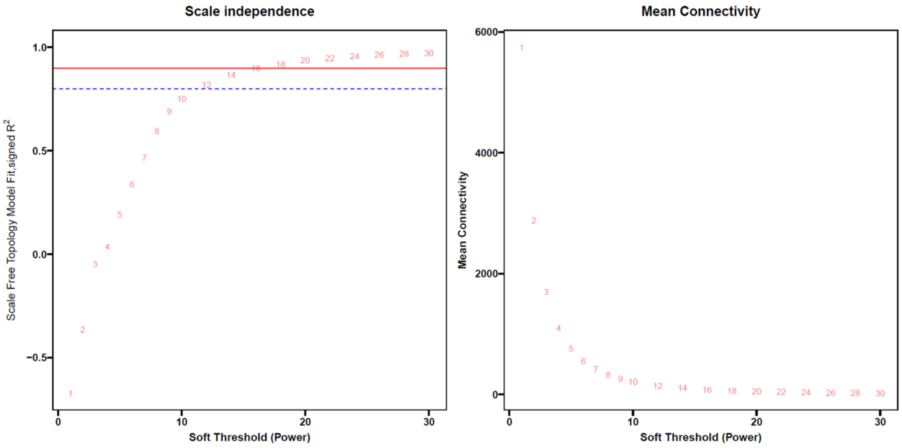

(B)

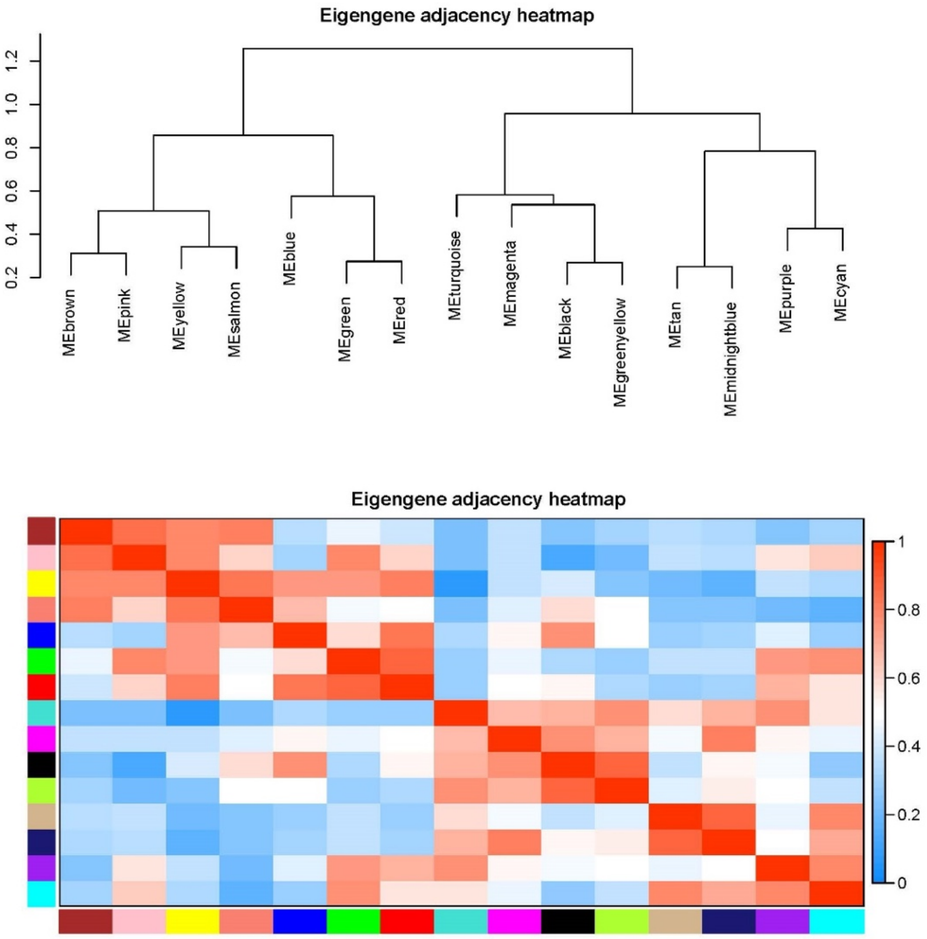

Supplementary Figure S16. (A) Soft threshold for WGCNA. **(B)** Eigengene adjacency heatmap.

(A)

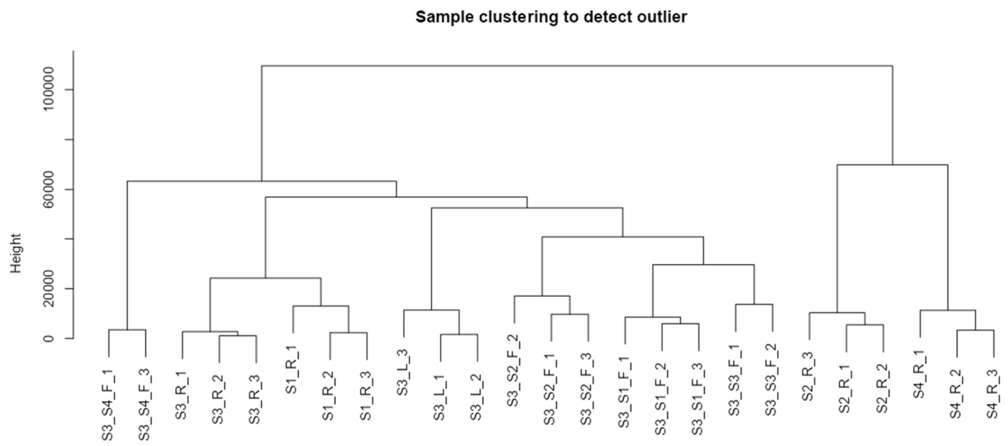

(B)

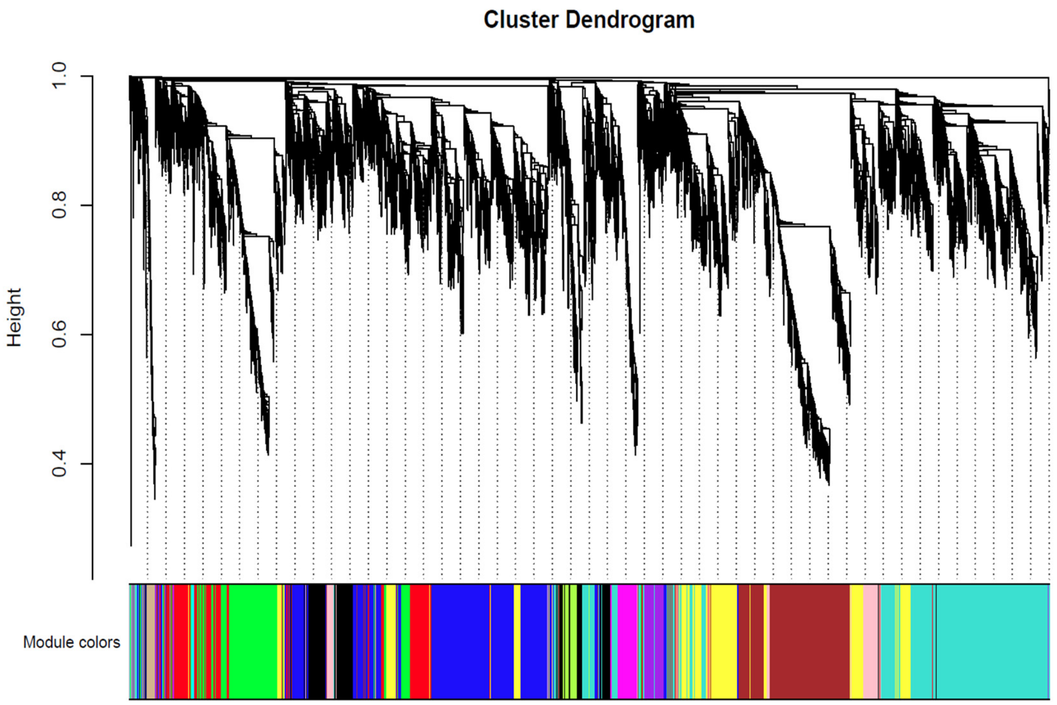

Supplementary Figure S17. (A) Sample clustering diagram. **(B)** Gene dendrogram obtained by hierarchical clustering with the module color indicated by the color of the row underneath. A total of 16 distinct modules were identified.

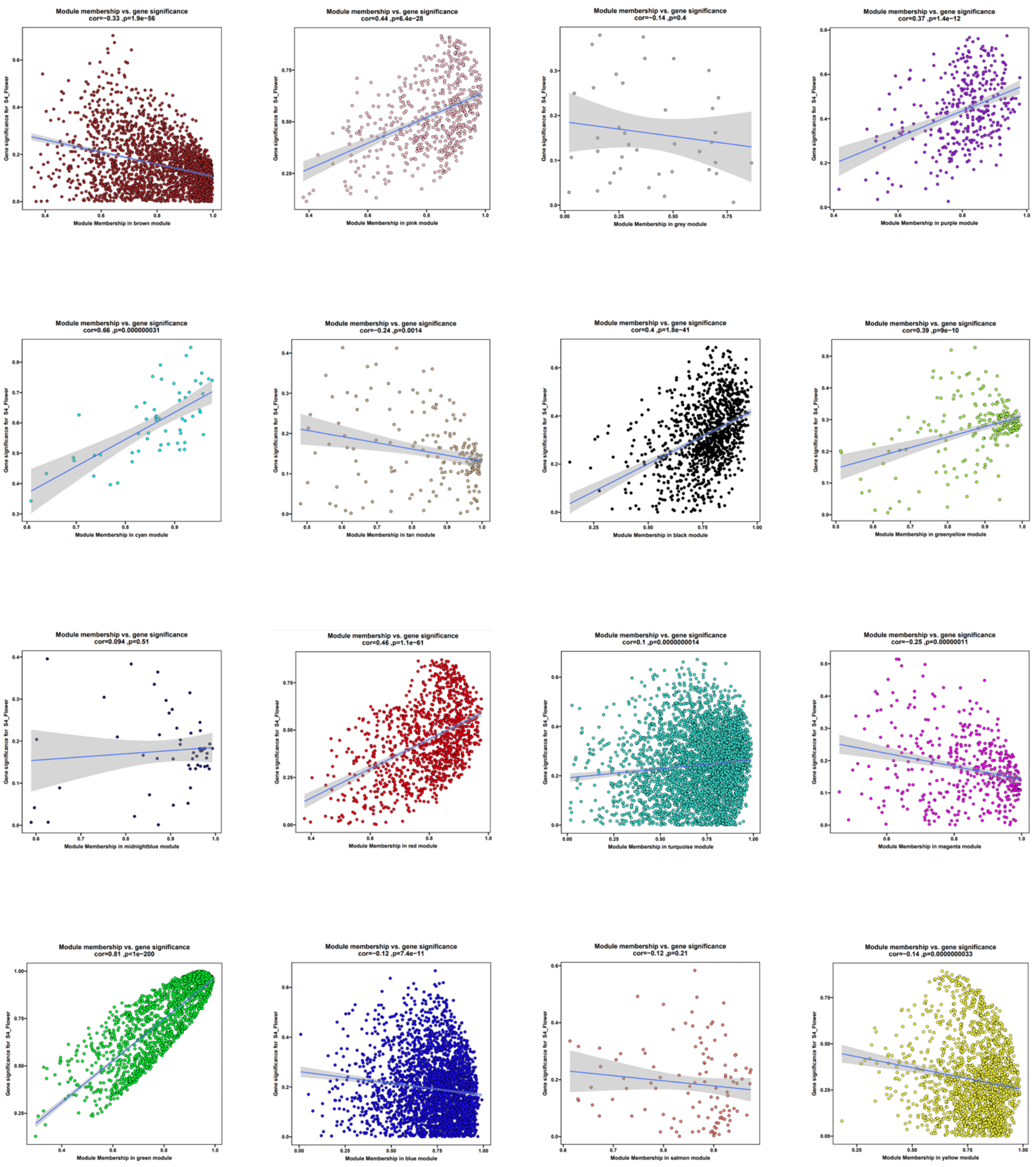

Supplementary Figure S18. Module membership vs. gene significance in each module from WGCNA.

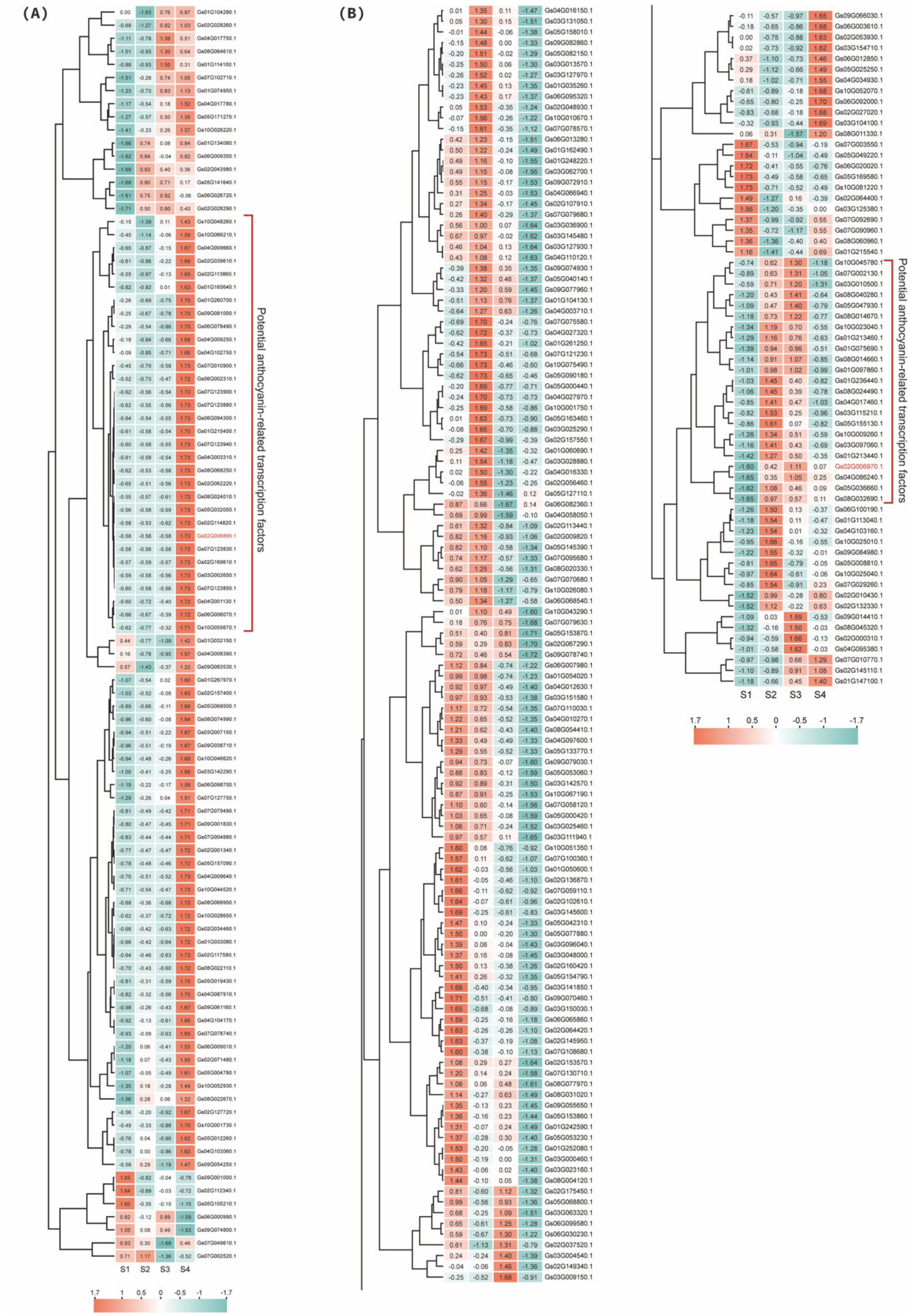

Supplementary Figure S19. (A) Heatmap showing the expression patterns of transcription factors (TFs) within the green module identified by WGCNA. **(B)** Heatmap of TFs in the blue module. Red-labeled gene names indicate putative MYB transcription factors potentially involved in the regulation of anthocyanin biosynthesis in *Gloriosa superba* tepals.
